# REPRODUCTIVE AGEING: Altered histone modification landscapes underpin defects in uterine stromal cell decidualization in aging females

**DOI:** 10.1530/REP-24-0171

**Published:** 2024-08-02

**Authors:** Laura Woods, Wendy Dean, Myriam Hemberger

**Affiliations:** 1 The Babraham Institute, Babraham Research Campus, Cambridge, United Kingdom; 2 Department of Cell Biology and Anatomy, Cumming School of Medicine, Hospital Drive NW, University of Calgary, Calgary, Alberta, Canada; 3 Alberta Children’s Hospital Research Institute, University of Calgary, Calgary, Alberta, Canada; 4 Department of Biochemistry and Molecular Biology, Cumming School of Medicine, Hospital Drive NW, University of Calgary, Calgary, Alberta, Canada

## Abstract

**In Brief:**

Advanced maternal age is associated with a higher rate of pregnancy complications that are unrelated to karyotypic abnormalities of the oocyte. This study shows that the murine uterine stroma undergoes profound epigenetic changes affecting active and repressive histone modification profiles that are associated with impaired endometrial functionality and underpin the decline in reproductive performance of aged females.

**Abstract:**

Decidualization describes the transformation of the uterine stroma in response to an implanting embryo, a process critical for supporting the development of the early embryo, for ensuring normal placentation and ultimately for a healthy reproductive outcome. Maternal age has been found to impede the progression of decidualization, heightening the risk of reproductive problems. Here, we set out to comprehensively characterize this deficit by pursuing transcriptomic and epigenomic profiling approaches specifically in the uterine stromal cell (UtSC) compartment of young and aged female mice. We find that UtSCs from aged females are globally far less responsive to the decidualization stimulus triggered by exposure to the steroid hormones estrogen and progesterone. Despite an overall transcriptional hyperactivation of genes that are differentially expressed as a function of maternal age, the hormonally regulated genes specifically fail to be activated in aged UtSCs. Moreover, even in their unstimulated ‘ground’ state, UtSCs from aged females are epigenetically distinct, as determined by genomic enrichment profiling for the active and repressive histone marks H3K4me3 and H3K9me3, respectively. We find that many hormone-inducible genes exhibit a profound lack of promoter-associated H3K4me3 in aged UtSCs, implying that a significant enrichment of active histone marks prior to gene stimulation is required to enable the elicitation of a rapid transcriptional response. With this combination of criteria, our data highlight specific deficits in epigenetic marking and gene expression of ion channels and vascular markers. These results point to fundamental defects in muscle-related and perivascular niche functions of the uterine stroma with advanced maternal age.

## Introduction

Embryo implantation and reproductive success depend on an adequate response of the uterus to the pregnancy hormones estrogen (E2) and progesterone (P4) (Adams & DeMayo 2015). The action of these steroid hormones ensures that the uterus acquires a receptive state in which the luminal epithelium is primed for embryo implantation (Wang & Dey 2006). In the mouse, implantation occurs around gestational day (E) 4.5 post fertilization when the embryo is at the expanded blastocyst stage. The physical stimulus of an implanting embryo triggers the uterine stroma to undergo a transformation process termed decidualization, during which the stromal cells cease to proliferate and become enlarged and often binucleated and polyploid (Das 2009). The physical stimulus of an implanting embryo can also be recapitulated by scratching of the uterine lining or by injection of oil or beads. The induced decidualization of uterine stromal cells (UtSCs) is critical to facilitate the early nurturing of the implanted embryo as well as to counteract excessive trophoblast invasion (Cha *et al.* 2012). Continuous and reciprocal crosstalk between the blastocyst’s outer trophectoderm cells and the decidualizing uterine stroma is central to the implantation process and, ultimately, to ensure normal placentation. Formation of a functional placenta is a milestone event for developmental progression which, in the mouse, occurs around mid-gestation at E10.5.

Decidualization occurs in two waves in the mouse, first in an area immediately surrounding the implanting blastocyst forming the primary decidual zone (PDZ) at E5.5–E6.5. During the following days, stromal cells adjacent to the PDZ continue to proliferate and then differentiate into decidual cells to establish the secondary decidual zone (SDZ) at E8.5 (Tan *et al.* 2002, Cha *et al.* 2012, Yuan *et al.* 2019). The importance of decidualization for reproductive success has been demonstrated in a series of mouse mutants in which this process is affected, often resulting in implantation failure or early post-implantation embryo demise (Mukherjee *et al.* 2006, Lee *et al.* 2007, Cha *et al.* 2012). The molecular processes driving decidualization are chiefly regulated by the pregnancy hormone P4. Deletion of the progesterone receptor (PGR) and specifically of the PGR-A isoform that confers P4’s function in the uterus exemplifies the importance of decidualization for reproductive success (Lydon *et al.* 1995, Mulac-Jericevic *et al.* 2000, 2003, Mulac-Jericevic & Conneely 2004, Wetendorf & DeMayo 2012). These knockout (KO) females are infertile and exhibit implantation failure and/or early post-implantation embryonic lethality. Furthermore, KO uteri fail to show a decidualization response of stromal cells upon traumatic stimulation and exhibit severe epithelial hyperplasia due to the unimpeded action of E2 (Mulac-Jericevic *et al.* 2000, Mulac-Jericevic & Conneely 2004).

Importantly, even in the absence of genetic mutations, the decidualization reaction may proceed inadequately, often with detrimental consequences on the developmental progression of embryogenesis (Lopes *et al.* 2009, Woods *et al.* 2017, Kokorudz *et al.* 2022). One prominent scenario that exacerbates these deficiencies is advanced maternal age (Biggers et al. 1962*b*, Talbert and Krohn 1966, Kong *et al.* 2012, Woods *et al.* 2017, 2020). Thus, it has been shown that C57BL/6 mouse females approaching the end of their reproductive lifespan, i.e. around 1 year of age, exhibit a blunted P4 responsiveness that results in a delay in decidualization by 1–2 days. As a consequence of these decidualization deficits, placental defects as well as embryonic abnormalities ensue (Woods *et al.* 2017, Kokorudz *et al.* 2022). Preimplantation-stage embryo transfers from aged females into young recipient females restore developmental progression to normal, thereby ruling out that the defects originate solely in the oocyte of the aged females (Woods *et al.* 2017). These data extend the impact of advanced maternal age beyond an exclusive focus on the increased frequency of karyotypic abnormalities in oocytes of older females to also include the critical impact of a deteriorating uterine environment (Woods *et al.* 2017).

The effects of advanced maternal age on decidualization are evident particularly at the level of PGR expression and in the stromal cell response to P4-regulated genes. Thus at E3.5, the normally high expression of PGR across all luminal epithelial cells appeared far more patchy and inconsistent in the uteri of aged females (Woods *et al.* 2017). Areas of high expression were adjacent to patches of luminal epithelial cells with little or no detectable PGR expression. While implantation rates themselves were not affected, a delay in SDZ formation was observed over subsequent days in development (Woods *et al.* 2017). Moreover, when UtSCs were isolated and subjected to *in vitro* decidualization through exposure to E2 and P4, a marked delay or failure in the activation of several selected P4-responsive candidate genes was observed (Woods *et al.* 2017). These data highlight a cell-intrinsic deficit of UtSCs from aged females to elicit an adequate transcriptional response in particular to the pregnancy hormone P4.

Transcriptional changes are fundamentally regulated by the epigenome. While DNA methylation is commonly thought of as conferring heritable instructions to gene expression through stable gene silencing, dynamic expression changes are modulated by histone modifications. Using whole uterine tissue, we have previously shown that advanced maternal age is associated with DNA hypermethylation at a few key loci that are required for decidualization to progress, such as the *Hoxa10*/*Hoxa11* locus (Benson *et al.* 1996, Rahman *et al.* 2006, Woods *et al.* 2020). The hypermethylated state at these loci correlates with the inability of the corresponding genes to acutely respond to the hormonal stimulus and to become transcriptionally upregulated (Woods *et al.* 2020). Yet the number of DNA hypermethylated loci was relatively small and unlikely to reflect the full spectrum of decidualization defects observed in aged females and, specifically, in aged UtSCs.

On this background, we sought to characterize the transcriptional changes that occur specifically in UtSCs as a function of age in much greater detail, to gain a deeper understanding of the age-induced changes before and after stimulation with pregnancy hormones. Moreover, we aimed at investigating the changes that occur at the histone modification level in UtSCs as a function of maternal age. We focused on two specific modifications, H3K4me3 and H3K9me3, as well-established active and repressive marks, respectively. We integrated these data with the RNA-seq data of stromal cells both before and after the induction of decidualization *in vitro*. Our data show that stromal cells of aged females are globally epigenetically distinct from those of young females, indicative of dysfunction that manifests as profound transcriptomic differences and an inability to undergo the finely tuned decidualization events required for a healthy reproductive outcome.

## Materials and methods

### Uterine endometrial stromal cells

UtSCs were isolated as previously described (Afonso *et al.* 2002, Woods *et al.* 2017). Mice used for UtSC recovery were C57BL/6*Babr* virgin females. ‘Young’ females were 8–16 weeks old, and ‘aged’ females were 48–54 weeks old, i.e. nearing the end of the reproductive lifespan. All animal experiments were conducted in full compliance with UK Home Office regulations and with the approval of the local animal welfare committee at The Babraham Institute and with the relevant project and personal licenses in place. Timed matings were set up with standard C57BL/6Babr stud males of 8–16 weeks of age, counting the morning of the vaginal plug as E0.5.

For stromal cell recovery, uteri from E3.5 mice were slit longitudinally and disaggregated with 2.5% pancreatin and 0.5% trypsin (type III) in Hank’s basic salt solution – calcium/magnesium-free (HBSS-CMF) – for 1.5 h on ice. The digested tissue was vortexed, and the medium was removed. The remaining tissue was washed twice in HBSS (discarding luminal epithelial cells) and incubated for 30 min at 37°C in HBSS containing 0.007% collagenase, 0.02% DNase, and 0.008% protease, with vortexing every 5 min. Dissociated tissues were then triturated, filtered through a 70 μm cell strainer (BD Biosciences), and spun at 1500 ***g*** for 5 min at 4°C. Cell pellets were resuspended in phenol-red-free DMEM: Nutrient Mixture F-12 (DMEM/F-12) (Thermo Fisher Scientific #21041-025) plus 10% fetal bovine serum (FBS), 1 mM sodium pyruvate (Thermo Fisher Scientific #11360-039), 1× antimycotic/antibiotic (Thermo Fisher Scientific #15240-062), and 50 µM 2-mercaptoethanol (Thermo Fisher Scientific #31350-010).

Isolated UtSCs were expanded by passaging three to four times and then subjected to the decidualization cocktail or vehicle only as a control. Decidualization was initiated by incubating the cells with complete DMEM/F-12 medium plus 10 nM β-estradiol (Sigma, #E2758), 1 µM medroxyprogesterone 17-acetate (Sigma, #M1629), and 10 µM 8-bromoadenosine 3′,5′-cyclic monophosphate (Sigma, #B5386) (E2+P4+cAMP). The culture medium was changed every 2 days with continuous supplementation with these treatments. Control untreated cells were cultured in the equivalent amount of vehicle (EtOH) alone.

All experiments were carried out in biological triplicates, i.e. on cells isolated from three individual mice per age group. Cells assessed by RNA-seq before and after hormone exposure were from the same set of mice and cell isolation procedure, handled in parallel. Cells for chromatin immunoprecipitation (ChIP)-sequencing were from a different set of mice, with the chromatin split in half for H3K4me3 and H3K9me3 pull-down, respectively.

### RNA extraction and RNA sequencing

Total RNA was prepared using the Allprep DNA/RNA Mini kit (Qiagen, 80204), followed by DNase treatment using the TURBO DNA-free kit (Life Technologies, AM1907), according to the manufacturers’ instructions. RNA-seq libraries were generated using the SureSelect Strand-Specific RNA library preparation kit (Agilent, G9691A), according to the manufacturer’s instructions. Libraries were quantified using the KAPA Library Quantification Kit (KAPA Biosystems, KK4824) and quality controlled on the Bioanalyzer 2100 system (Agilent). Indexed libraries were pooled and sequenced with a 100 bp single-end protocol on an Illumina HiSeq2500 sequencer. Fastq data were trimmed for adaptors using Trim Galore! v0.4.4 before mapping to the *Mus musculus* GRCm38 genome assembly using Hisat2 v2.1.0.

### Chromatin immunoprecipitation sequencing for histone modifications

Histone ChIP was performed on pellets of 200,000 UtSCs isolated from E3.5 uteri from young (*n* = 3) and aged (*n* = 3) mice, snap-frozen at passage 3–4. Frozen pellets were resuspended in 10 mM Tris pH 7.5, 10 mM NaCl, 3 mM MgCl_2_, 1 mM CaCl_2_, and 0.4% NP-40, before separating into three equal fractions. All buffers were treated with protease inhibitors (Roche, 5056489001) to prevent the degradation of histones. Chromatin was digested with micrococcal nuclease (MNase; Sigma, N5386-200UN) at 1×, 1/2×, and 1/4× concentrations for 8 min at 37°C. The digest was quenched on ice with EDTA to a final concentration of 10 mM, before centrifuging at 2500 ***g*** at 4°C. The supernatant (S1) was isolated, and the pellet was resuspended in 10 mM Tris pH 7.5, 10 mM NaCl, 3 mM MgCl_2_, 0.25 mM EDTA, and 0.4% NP-40 and incubated on ice for 15 min. The resuspended pellet was passed four times through a 20-gauge needle and four times through a 25-gauge needle, before centrifuging at 10,000 ***g*** for 10 min a 4°C and combining the supernatant (S2) with S1. The NaCl concentration was raised to 150 mM before incubating on ice for 20 min and centrifuging at 10,000 ***g*** for 15 min and retaining the supernatant. About 20 µL chromatin was taken, and DNA was purified using the QIAquick PCR purification kit (Qiagen, 28106) and run on an Agilent BioAnalyzer 2500 to confirm the extent of chromatin digestion. Chromatin from the three digests was pooled.

The digested chromatin was diluted to a final volume of 800 μL per ChIP. About 20 μL of Dynabeads Protein A for Immunoprecipitation beads (Dynabeads 10001D) per ChIP were washed 3× with 10 mM Tris pH 7.4, 50 mM NaCl, 5 mM EDTA (NChIP buffer), and 10 μL beads were added to 800 μL of chromatin and 1 μg rabbit IgG (Santa Cruz Biotechnology, sc-2027X) and rotated for 2 h at 4°C to preclear chromatin. Beads were discarded, and 10% of the chromatin was taken for input before adding 0.25 μg of antibodies against H3K4me3 (Abcam, ab8580), H3K9me3 (Abcam, ab8898), or rabbit IgG (Santa Cruz Biotechnology, sc-2027X) to the precleared chromatin and rotating overnight at 4°C. About 10 μL of prewashed protein A beads were added to each ChIP and rotated for 4 h at 4°C. After 4 h, the beads were washed five times in ice-cold NChIP buffer, then resuspended in NChIP + 1% SDS at room temperature for 15 min, and treated with TE buffer pH 6.5 and proteinase K for 30 min at 45ºC. Input and ChIP samples were purified using the QIAquick PCR purification kit (Qiagen, 28104). qPCR was performed for regions known to be enriched or depleted for H3K4me3 and K3K9me3 to check the efficiency of the pull-down.

DNA libraries were generated from H3K4me3, H3K9me3, and input DNA using the NEBNext Ultra II DNA library prep kit (New England Biolabs, E7645), following the manufacturer’s instructions. Libraries were purified and double size-selected using Agencourt AMPure XP beads (Beckman Coulter, A63881), and amplified with the program 98°C for 30 s, 12–15 cycles of 98°C for 10 s, 65°C for 75 s, followed by a final extension at 65°C for 5 min. ChIP libraries were purified with AMPure XP beads. Libraries were assessed for quality using both the BioAnalyzer 2100 System (Agilent) and quantified using the KAPA Library Quantification Kit (KAPA Biosystems, KK4824). Indexed libraries were pooled and sequenced on an Illumina HiSeq2500 sequencer, using a 50 bp paired-end protocol. Raw fastq data were mapped to the *M. musculus* GRCm38 genome assembly using Bowtie v2.

### Bioinformatic analysis

Data were quantitated using the RNA-seq quantitation pipeline in SeqMonk software (http://www.bioinformatics.babraham.ac.uk) and normalized according to total read count (reads per million mapped reads, RPM). Differential expression was calculated using DESeq2 with *P* < 0.05 and adjusted for multiple testing correction using the Benjamini–Hochberg method and for stringent difference analysis including a value difference filter of Log_2_ ±2 (i.e. four-fold higher or lower expression) in SeqMonk. Most graphs, including heatmaps, box whisker plots, and Star Wars plots, were generated in SeqMonk. Principal component analysis (PCA) was performed on all expressed genes or on all genes in 5 kb proximity to differentially DNA-methylated regions in young and aged uteri (Woods *et al.* 2020). Gene Ontology (GO) analyses were performed on the list of genes found to be significantly differentially expressed (DE), using DAVID (Huang da *et al.* 2009), WebGestalt (Wang *et al.* 2017), enrichR (Kuleshov *et al.* 2016), and Metascape (Zhou *et al.* 2019) resources. For the analysis of gene proximity to PGR- and ESR1-binding elements, genes DE between young and aged stromal cells were screened for 5 kb proximity to PGR and ESR1 ChIP-seq elements (Hewitt *et al.* 2012, Rubel *et al.* 2012, Gertz *et al.* 2013). H3K4me3 and H3K9me3 ChIP-seq peaks were called using the MACS peak caller function in SeqMonk. Peaks containing significantly differentially enriched levels were identified using DESeq2 with *P* < 0.05, as well as with the LIMMA statistical test in SeqMonk. For H3K4me3, consensus peaks were called as those regions that overlapped between these two statistical tests. For H3K9me3, only the DESeq2 enrichment was used for consensus peak calling. Plots of gene expression data were generated in GraphPad Prism v10.2.2. Statistical significance analysis was performed by a two-way ANOVA test.

### Data availability

High-throughput sequencing data have been deposited in Gene Expression Omnibus under accession numbers GSE138375 (RNA-seq) and GSE267479 (ChIP-seq).

## Results

### Transcriptomic differences between uterine stromal cells from young and aged females

**Figure 1:**
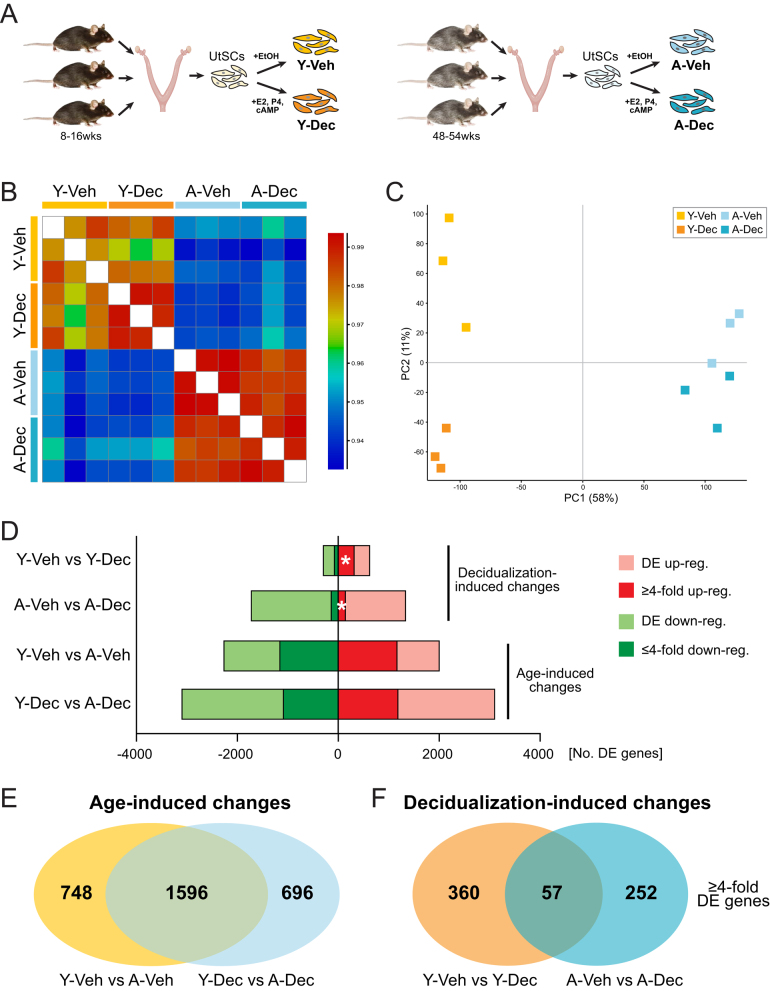
Transcriptomic profiling of endometrial uterine stromal cells from young and aged females. (A) Experimental design of uterine stromal cell (UtSC) isolation and culture from young (‘Y’) and aged (‘A’) virgin C57BL/6 female mice. UtSCs from each mouse were either induced to decidualize by exposure to E2, P4, and cAMP for 4 days (‘Y-Dec’, ‘A-Dec’) or treated with vehicle only (ethanol = EtOH) for the same duration (‘Y-Veh’, ‘A-Veh’). (B) Heatmap of Euclidean distances displaying the overall degree of sample similarity. (C) Principal component analysis (PCA) of global transcriptomes from young and aged UtSCs before and after pregnancy hormone exposure. (D) Number of differentially expressed (DE) genes as determined by DESeq2 analysis in the pairwise comparisons of sample groups, as indicated. Four-fold up- and down-regulated genes are highlighted by the darker colors. The white asterisks denote the stark and particularly important difference in the number of robustly (≥ four-fold) up-regulated genes between young and aged UtSCs for the decidualization-induced changes. (E) Overlap in DE genes with ≥ four-fold expression differences as a function of age, i.e. those genes identified in both the comparisons of Y-Veh vs A-Veh and Y-Dec vs A-Dec. (F) Overlap in DE genes with ≥ four-fold expression differences as a function of decidualization, i.e. those genes commonly identified in Y-Veh vs Y-Dec and A-Veh vs A-Dec.

To identify transcriptional differences in UtSCs before and after *in vitro*-induced decidualization, we isolated UtSCs by enzymatic digestion of uterine tissue from young (8–16 weeks) and aged (48–54 weeks) virgin C57BL/6 females. UtSCs were plated in phenol red-free medium, passaged, and then collected for RNA in the presence of vehicle alone (Veh) and upon exposure to E2, P4, and cAMP for 4 days (Dec), a well-established treatment regimen to induce decidualization (Fig. 1A) (Mazur *et al.* 2015, Feng *et al.* 2017, Woods *et al.* 2017). This experimental layout ensured that decidualization responses could be monitored in UtSCs from individual mice (i.e. paired samples), thereby accounting for potential biological variability. We performed these experiments on three independent mice from each age group (Fig. 1A).

Comparing the overall transcriptomic similarities across all genes revealed that the largest difference in expression profiles was introduced by maternal age. This can be appreciated both in a heatmap of Euclidean distances and in PCA plots, in which age accounts for 58% of transcriptional differences along PC1 (Fig. 1B and C). Interestingly, however, while UtSCs from young mice exhibit a clear separation between untreated ‘ground state’ and decidualized samples along PC2 (Fig. 1C), this hormonal induction-driven difference is much less pronounced in UtSCs from aged females. This lack of hormone responsiveness can be appreciated by aged cells exhibiting a high degree of transcriptional similarity between samples with and without hormone exposure (Fig. 1B and C). These data highlight a profound loss in the capacity to undergo hormonally induced transcriptional changes in UtSCs as a function of advanced maternal age on a global level.

### Age-induced vs hormone-stimulated transcriptional differences

We then performed DESeq2 analysis to identify DE genes between all four sample groups, i.e. UtSCs from young females before (Y-Veh) and after (Y-Dec) decidualization, as well as UtSCs from aged females subjected to the same treatment regimens (A-Veh, A-Dec) (Fig. 1A). We identified DE genes in pairwise comparisons between these groups, separated them into up- and down-regulated genes, and also highlighted more extreme expression changes of genes with four-fold differences in transcript levels (Log_2_ RPM ≥ ±2) (Fig. 1D). As expected, the highest number of DE genes was identified in the comparison of young vs aged samples, i.e. in Y-Veh vs A-Veh (4294 genes) and in Y-Dec vs A-Dec (6222 genes). We broadly termed these genes ‘age-induced changes’. Overall, there were similar numbers of up- and down-regulated genes in these comparisons (Y-Veh vs A-Veh: 2015 up, 2279 down; Y-Dec vs A-Dec: 3112 up, 3110 down). The number of DE genes was higher in the Dec group, reflecting the lack of hormone responsiveness in the aged cells.

Comparing the Veh vs Dec samples within each age group identified genes that were DE as a function of decidualization. Due to the tighter sample grouping and reduced statistical variation, the nominal number of DE genes was higher in the A-Veh vs A-Dec comparison (3081 genes) as opposed to the Y-Veh vs Y-Dec cells (946 genes) (Fig. 1D). However, the more meaningful differences were revealed in the group of genes with four-fold expression differences, where a substantially higher number of genes were found up-regulated in the young compared to the aged cells (Y: 329/946 (35%) genes ≥ four-fold up, A: 156/3081 (5%) genes ≥ four-fold up) (Fig. 1D, white asterisks). These data identify that the transcriptional up-regulation of decidualization genes in response to pregnancy hormones is disproportionately affected with age.

Overlap between the lists of DE genes from the pairwise comparisons identified a robust cohort for the age-induced changes, with 52.5% (1596/3040) of four-fold DE genes shared between Veh and Dec (Fig. 1E). By comparison, the overlap in hormone exposure-induced transcriptional changes was relatively minimal between young and aged cells, with only 8.5% (57/669) of shared ≥ four-fold DE genes (Fig. 1F). These data underpin the notion that maternal age causes the most profound and consistent changes in transcriptional output in UtSCs regardless of differentiation state, and that the induction of decidualization is severely perturbed in aged UtSCs on the global level.

### Age-induced transcriptional changes affect cell adhesion and angiogenesis pathways

**Figure 2:**
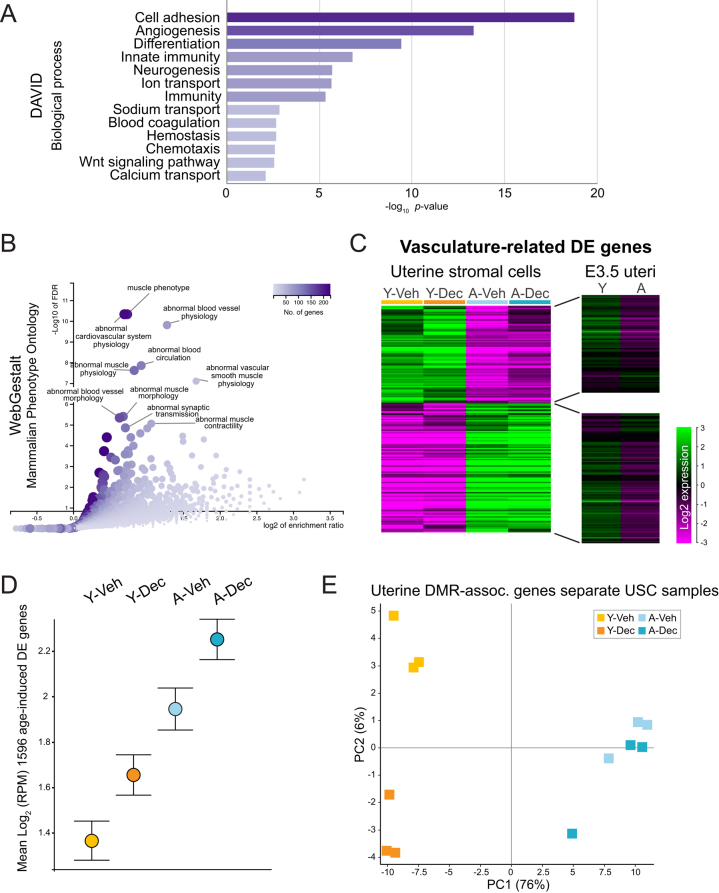
Age-induced DE genes relate to vascular and muscle biology. (A) GO analysis of the 1596 common age-related DE genes from Fig. 1E, focusing on the biological process enrichment terms in DAVID (Huang da *et al.* 2009). (B) GO analysis of the same set of genes, focusing on mammalian phenotype ontology terms in WebGestalt (Wang *et al.* 2017). (C) Heatmap of expression levels of the 186 DE genes contained within the enrichment term ‘blood vessel development’ (GO: 0001568) as determined by Metascape (Zhou *et al.* 2019). Genes are separated into up- and down-regulated groups in young and aged UtSCs, and their expression levels in E3.5 uterine tissues are plotted on the same scale (Woods *et al.* 2017). (D) ‘Star Wars’ plot of mean gene expression levels of the 1596 DE genes that change as a function of age. Overall transcriptional output of these genes is elevated with decidualization but in particular with age. (E) PCA plot on RNA-seq data of DE genes associated with differentially DNA methylated regions in E3.5 uteri of young and aged females (Woods *et al.* 2020).

Focusing on the 1596 common age-induced transcriptional changes in UtSCs, we performed GO analysis using multiple web tools including enrichR, DAVID, WebGestalt, and Metascape (Huang da *et al.* 2009, Kuleshov *et al.* 2016, Wang *et al.* 2017, Zhou *et al.* 2019). Consensus-enriched pathways within this set of DE genes were related to cell adhesion, extracellular matrix composition, muscle phenotypes, as well as to angiogenesis and blood vessel morphology (Fig. 2A and B). The cell adhesion and extracellular matrix-related changes likely reflect the well-documented reorganization of extracellular matrix components in aged uteri, such as increased infiltration with collagen fibers (Biggers et al. 1962*a*).

As to the vascular gene expression differences, the UtSCs isolated from aged mice exhibited higher expression levels for both arterial and venous marker genes, including Delta-like canonical Notch ligand* Dll1*, Notch receptors *Notch1* and *Notch4*, gap junction protein alpha 4 (*Gja4*, a.k.a. connexin 37), C-X-C motif chemokine receptor* Cxcr4*, activin A receptor type II-like 1 (*Acvrl1*), BMX non-receptor tyrosine kinase (*Bmx*), as well as Eph receptor B4 (*Ephb4*) and neuropilin 2 (*Nrp2*), respectively. Yet the vast majority of pericyte markers, including melanoma cell adhesion molecule (*Mcam*, a.k.a. CD146) and chondroitin sulfate proteoglycan 4 (*Cspg4*, a.k.a. NG2), remained unchanged (Kirkwood *et al.* 2021). Platelet-derived growth factor B (*Pdgfb*), a growth factor produced by smooth muscle cells that leads to the establishment of a thicker media, was dominantly expressed in aged UtSCs. These changes in vascular marker gene expression may reflect the deregulation of gap junction protein alpha 1 (*Gja1*), the gene encoding connexin 43, which is involved in the regulation of decidual angiogenesis (Winterhager *et al.* 2013, Massri *et al.* 2023). *Gja1* is down-regulated in aged UtSCs as well as with hormone treatment, and this reduced expression level may indeed cause the up-regulation of pro-angiogenic genes such as FMS-like tyrosine kinase 1 (*Flt1*). Lymphatic markers SRY-box 18 (*Sox18*), FMS-like tyrosine kinase 4 (*Flt4*), prospero homeobox 1 (*Prox1*), and lymphatic vessel endothelial hyaluronan receptor 1 (*Lyve1*) were all highly enriched in aged UtSCs (Wolf *et al.* 2019). Fibroblast growth factor 2 (*Fgf2*), a growth factor normally expressed in undifferentiated cells underneath the myometrium which plays a critical role both in the regulation of angiogenesis and in decidualization (Paria *et al.* 2001, Liu *et al.* 2022), was dramatically down-regulated in aged UtSCs, irrespective of hormone treatment.

To assess these changes in vascular gene expression more systematically, we identified 186 factors out of the common 1596 age-induced DE genes that determined the enrichment term ‘blood vessel development’ (GO: 0001568) in Metascape. Hierarchical clustering of these genes showed that approximately half of them were up- and down-regulated in the aged UtSCs, respectively (Fig. 2C). Interestingly, however, when interrogating these same genes against E3.5 uterine tissue (Woods *et al.* 2017), they did not exhibit the same dramatic expression differences in uteri from young and aged female mice (Fig. 2C). These data suggest that the developmental capacity of UtSCs has changed with maternal aging, making them more susceptible to transcriptional dysregulation. Based on the exacerbated effects *in vitro*, they are potentially more prone to misguided gene expression and differentiation depending on the growth environment. In any case, the data are indicative of an impaired angiogenesis and of functional deficits in stromal cells, changes that have also been observed in mouse mutant models that suffer from decidualization defects (Yuan *et al.* 2019, Massri *et al.* 2023).

We then compared the mean transcriptional output of the 1596 commonly deregulated genes with age and found that, overall, average expression levels increased with age as well as with decidualization (Fig. 2D). These data indicate that the total number of transcripts increased upon hormonal stimulation. In addition, and perhaps more importantly, maternal age resulted in higher mean expression levels, indicative of a loss in the fidelity of transcriptional priming.

### DNA methylation changes in the uterus correlate with functionally relevant transcriptional changes in UtSCs

Next, we integrated the transcriptomes of the young and aged UtSCs with the DNA methylation data of uterine tissue from females of the same age groups, a study we had conducted earlier (Woods *et al.* 2020). We confirmed that genes that we had found hypermethylated in aged uteri, such as homeobox A10 (*Hoxa10*), homeobox A11 (*Hoxa11*) and frizzled class receptor 2 (*Fzd2*), also exhibited lower expression levels in aged UtSCs (Supplementary Figure 1, see section on supplementary materials given at the end of this article). These data corroborated our earlier findings but, importantly, pinned them to a specific uterine compartment, the stromal cells.

When focusing on genes associated with differentially methylated regions (DMRs) in aged uteri, we found that 37 out of 78 (47%) of these loci were also DE between young and aged UtSCs. Performing a PCA on these 37 DMR-associated genes separated the young and aged UtSCs depending on age and decidualization state (Fig. 2E), an output that appeared very similar to that obtained with the global UtSC transcriptomes (Fig. 1C). This finding indicated that differentially methylated loci identified in whole uteri translated into meaningful expression differences in UtSCs and that the impact of aging could be detected on this small subsample of genes alone.

### Profound decidualization deficits in aged UtSCs

**Figure 3:**
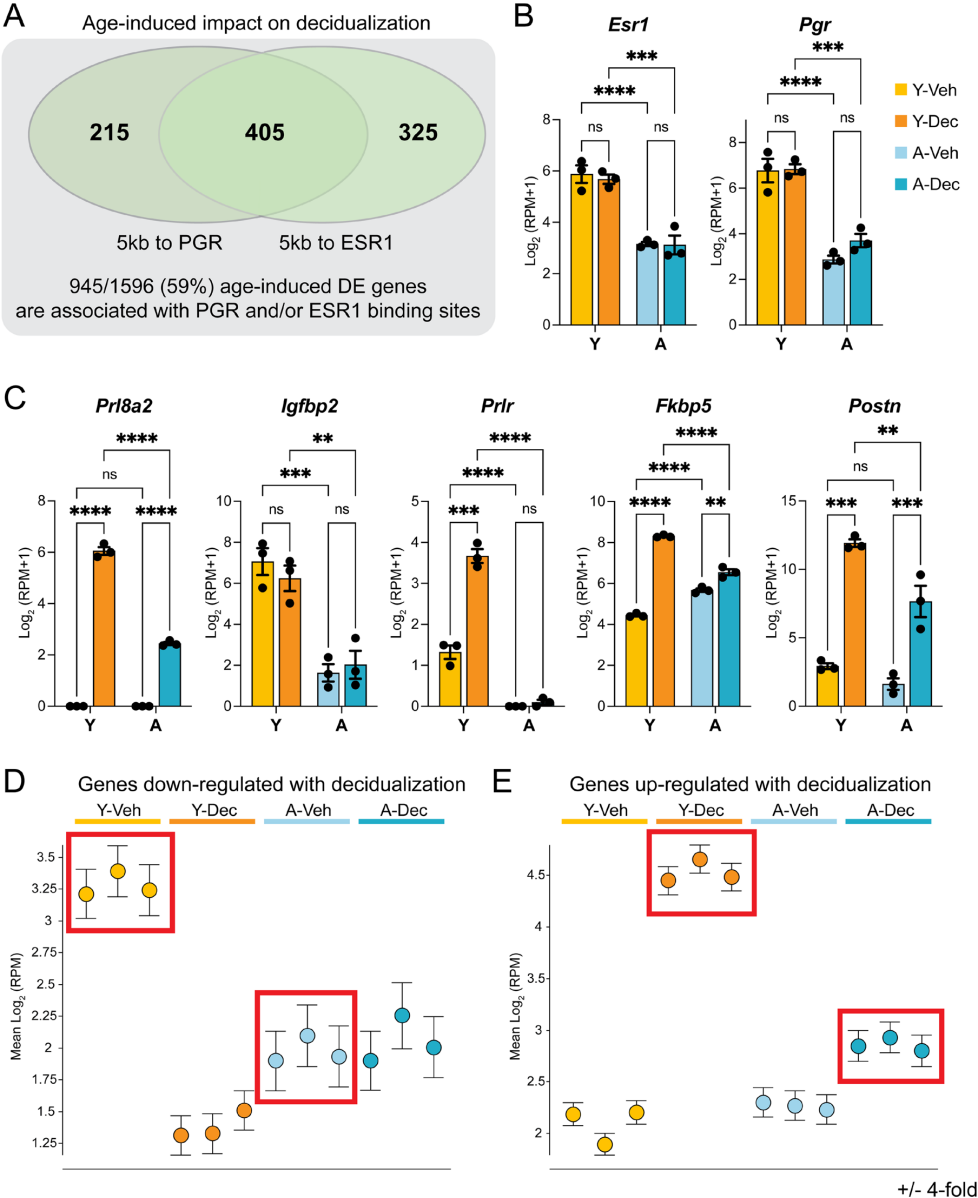
RNA-seq analysis reveals profound decidualization defects in aged UtSCs. (A) Association of 1596 common age-induced DE genes with estrogen receptor (ESR1) and progesterone receptor (PGR) binding sites in the genome. About 59% of DE genes are within 5 kb of an ESR1 and/or PGR-binding element. (B) Reduced expression of *Esr1* and *Pgr* in aged UtSCs, plotted as Log_2_(RPM+1) expression values from the RNA-seq data. Statistical significance analysis was performed by two-way ANOVA tests. (C) Mis-expression of various important decidualization genes as a function of age. Bar color identity conforms to the same legend as in B. Statistical significance analysis was performed by two-way ANOVA tests. (D) Assessment of mean gene expression changes for loci that are normally down-regulated upon pregnancy hormone exposure in young UtSCs (Y-Veh vs Y-Dec, four-fold DE). These genes are already expressed at far lower levels in untreated aged UtSCs (red rectangles). (E) Mean gene expression changes of loci that are normally up-regulated upon pregnancy hormone exposure in young UtSCs (Y-Veh vs Y-Dec, four-fold DE). These genes broadly fail to be induced to adequate levels in aged UtSCs (red rectangles).

We had previously described a delay in the progression of decidualization in UtSCs from aged females based on a few hallmark genes (Woods *et al.* 2017). In the current study, we expanded this analysis by characterizing the global transcriptomic changes in UtSCs as a function of maternal age. Interestingly, we found that almost 60% of age-induced DE genes (945/1596) were close (within 5kb) to estrogen receptor (ESR1) and/or PGR binding sites in the genome (Fig. 3A), a dramatic enrichment over background (38%). Affected genes included *Pgr* and *Esr1* themselves, for which overall reduced expression levels were observed in the aged samples (Fig. 3B), in line with previously reported immunostaining data on whole uteri (Woods *et al.* 2017). Contrary to what is normally observed *in vivo*, *Pgr* was not up-regulated upon induction of decidualization in young UtSCs. However, we observed a robust up-regulation of other well-known decidualization markers including prolactin family 8 subfamily a member 2 (*Prl8a2*), insulin-like growth factor-binding protein 2 (*Igfbp2*), prolactin receptor (*Prlr*), FK506-binding protein 5 (*Fkbp5*), and periostin (*Postn*) in Y-Dec compared to Y-Veh samples (Fig. 3C). Intriguingly, this cohort of genes exhibited a distinct lack of activation in the aged decidualized samples, sometimes combined with reduced basal expression levels (Fig. 3C). These data exemplify the profound failure of aged UtSCs to elicit an adequate decidualization response.

To investigate these age-induced decidualization defects in more detail, we specifically focused on genes that were hormonally regulated in young UtSCs (DE in Y-Veh vs Y-Dec, Log_2_ ≥ ±2). Separating these genes into up- and down-regulated groups and plotting mean expression levels across all samples revealed two striking findings: first, the baseline expression of UtSC genes that were down-regulated upon hormone exposure in young cells exhibited reduced mean expression levels in untreated aged UtSCs (Fig. 3D, red rectangles). Thus, despite the increased mean transcriptional output observed for the shared aging genes (Fig. 2D), this specific group of decidualization-sensitive genes exhibited a striking transcriptional dysregulation in uninduced UtSCs of aged females. Secondly, and perhaps more importantly, genes that were normally robustly activated upon hormone exposure displayed a markedly blunted response in aged UtSCs on a global scale (Fig. 3E). This gene set illuminates the lack of hormone responsiveness and the failed decidualization capacity of aged UtSCs.

### UtSCs of young and aged females are epigenetically distinct

**Figure 4:**
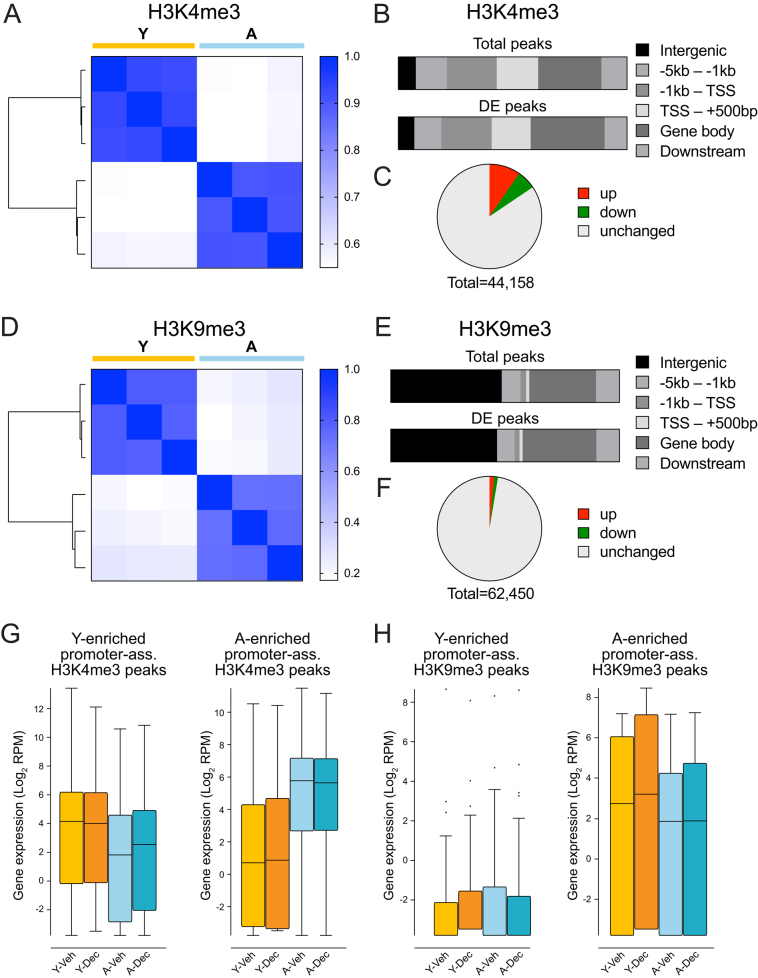
Epigenetic profiling of UtSCs from young and aged females for H3K4me3 and H3K9me3. (A) Heatmap of read count enrichment under peaks for the 6702 differentially enriched H3K4me3 peaks between young and aged UtSCs. (B) Genomic distribution of all, as well as of differentially enriched, H3K4me3 peaks relative to position around genes. (C) Pie chart of differentially enriched H3K4me3 peaks. About 15.5% of all H3K4me3 peaks display statistically significant differential enrichment between young and aged UtSCs. (D) Heatmap of read count enrichment under peaks for the 1649 differentially enriched H3K9me3 peaks between young and aged UtSCs. (E) Genomic distribution of all, as well as of differentially enriched, H3K9me3 peaks relative to position around genes. (F) Pie chart of differentially enriched H3K9me3 peaks. 2.6% of all H3K9me3 peaks display statistically significant differential enrichment between young and aged UtSCs. (G) Box whisker plots of RNA-seq data on genes enriched for promoter-associated H3K4me3 in young and aged UtSCs. Promoter H3K4me3 enrichment is clearly associated with elevated gene expression levels. (H) Box whisker plots of RNA-seq data on genes enriched for promoter-associated H3K9me3 in young and aged UtSCs. Due to the few sites identified, genes whose promoters are enriched for H3K9me3 in young UtSCs do not exhibit overtly changed expression levels; however, gene expression levels of loci whose promoters are enriched for H3K9me3 in aged UtSCs do overall exhibit lower expression levels. Promoters in G and H were defined as −1 kb to +200 bp surrounding the transcriptional start site.

Our next goal was to characterize the epigenetic landscape of young and aged UtSCs for the active and repressive histone marks H3K4me3 and H3K9me3, respectively. We were specifically interested to see whether UtSCs from young and aged females differed *a priori* in the absence of the hormonal stimulus, i.e. whether the epigenome might contribute to preventing an adequate decidualization response. For this reason, we performed ChIP-seq profiling on untreated UtSCs isolated from young and aged females, equivalent to the samples used for RNA-seq. We identified 44,158 and 62,450 total peaks for H3K4me3 and H3K9me3, respectively. Importantly, differential enrichment analysis clearly separated young from aged UtSCs for both marks, revealing profound epigenetic differences between UtSCs from young and aged females (Fig. 4A and D). As expected, peak distribution for H3K4me3 was centered around the transcriptional start sites (TSS) of genes, whereas H3K9me3 exhibited a broader genomic distribution (Fig. 4B and E, Supplementary Figure 2). The genomic distribution of differentially enriched peaks broadly recapitulated the same pattern as that of all peaks, demonstrating that the differentially enriched peaks were not preferentially localized to unique subsets of genomic regions, as defined by distance relative to genes (Fig. 4B and E). The number of differentially enriched peaks was far greater for H3K4me3 compared to H3K9me3 (Fig. 4C and F), a finding in line with the observed global transcriptional changes that are reflected chiefly by promoter H3K4me3 enrichment. Moreover, more H3K4me3 peaks were gained than lost in aged UtSCs, a finding that corroborates the aberrant transcriptional hyperactivation observed at age-related genes (Fig. 2D and 4C).

When grouping the promoter-associated differentially enriched histone modification peaks into those that were elevated in young or aged samples, a clear correlation with transcriptional output of the corresponding genes was observed for H3K4me3 (Fig. 4G). Thus, genes whose promoters were more highly H3K4me3-enriched in young UtSCs were also more highly expressed in young samples; this correlation was even clearer for genes that gained H3K4me3 in aged samples, again pointing to the strong gain in transcriptional activation in aged UtSCs. Due to the small number of H3K9me3-enriched peaks at gene promoter regions, the reverse correlation, i.e. higher H3K9me3 enrichment and lower gene expression, was not evident for the few young-enriched sites, but it could be made out for the age-enriched H3K9me3-marked genes (Fig. 4H).

### Inadequate H3K4me3 priming correlates with failing activation of decidualization genes

**Figure 5:**
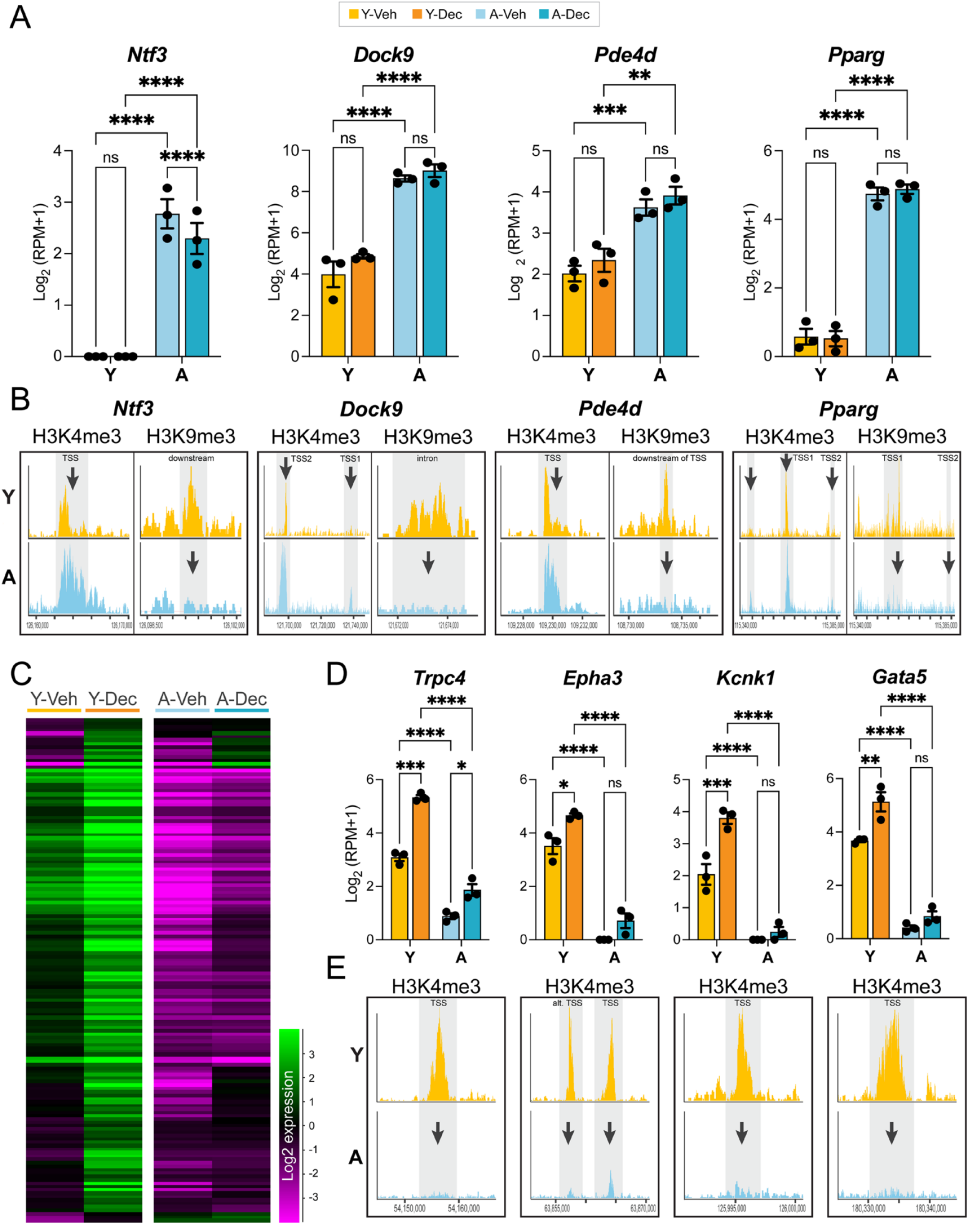
Hormone-responsive genes are (pre-)determined by high H3K4me3 enrichment. (A) Gene expression changes in young and aged UtSCs before or after hormonal stimulation of loci that are differentially enriched for H3K4me3 and H3K9me3. Statistical significance analysis was performed by a two-way ANOVA test. (B) Read count enrichment of H3K4me3 and H3K9me3 peaks in young and aged UtSCs at the corresponding genes. Peaks significantly depleted in enrichment are shaded and marked by an arrow. Peak locations relative to the gene are indicated. (C) Heatmap of genes that are DE between untreated and treated young UtSCs (Y-Veh vs Y-Dec) and that are associated with higher H3K4me3 peaks in young UtSCs. All of these genes become up-regulated upon hormone exposure in young UtSCs. All of these genes are also expressed at significantly reduced levels in aged UtSCs. (D) Examples of such hormone-responsive but age-deficient genes. Importantly, these genes functionally relate to ion channel function and peri-vascular niche markers. Bar color identity conforms to the same legend as in A. Statistical significance analysis was performed by two-way ANOVA tests. (E) H3K4me3 enrichment at the promoters of these same genes. Failing transcriptional activation correlates with severe depletion of H3K4me3 at the promoters of these genes in aged UtSCs.

We then investigated the genes identified as DE as well as differentially marked by H3K4me3 and H3K9me3 in more detail. This analysis revealed that around 50% of mis-expressed genes in ground state UtSCs (Y-Veh vs A-Veh) were also differentially marked by histone modifications, in particular H3K4me3. This included a number of genes that are not normally expressed in UtSCs, such as neurotrophin 3 (*Ntf3*), or that are profoundly over-expressed upon aging, such as dedicator of cytokinesis 9 (*Dock9*), phosphodiesterase 4D (*Pde4d*), and peroxisome proliferator-activated receptor gamma (*Pparg*), where enrichment of H3K4me3 and depletion of H3K9me3 correlated with elevated gene expression in aged UtSCs (Fig. 5A and B).

Our particular focus, however, was drawn to the group of decidualization genes, i.e. those genes that are DE in young UtSCs upon hormone exposure (Y-Veh vs Y-Dec). Of these 946 genes, 149 were marked with a Young-enriched H3K4me3 peak in undifferentiated conditions (Supplementary Table 1). Assessing the expression dynamics of these 149 genes demonstrated that all of them were even more highly expressed upon the induction of decidualization in young cells (Fig. 5C). This is a remarkable observation as the parameters *per se* did not select for genes that change in expression in this particular direction. Moreover, all of these genes failed to be adequately induced in aged UtSCs (Fig. 5C and D). This group of genes contained important factors, some of which have been previously associated with the progression of decidualization, such as the Ca^2+^ channel *Trpc4* (transient receptor potential cation channel, subfamily C, member 4) (De Clercq *et al.* 2015). Other examples included genes encoding for the Eph receptor A3 (*Epha3*), the potassium channel *Kcnk1* (potassium channel, subfamily K, member 1), and the transcription factor *Gata5* (GATA binding protein 5) (Fig. 5D). All of these genes were hormone-responsive and up-regulated upon decidualization in young UtSCs (Fig. 5D); they were also strikingly depleted in H3K4me3 abundance in aged UtSCs, even in undifferentiated conditions (Fig. 5E). These epigenetic profiles suggest that the up-regulation of this particular subset of genes requires a considerable enrichment in H3K4me3 already in undifferentiated UtSCs, which permits the gene loci to elicit an acute transcriptional response to the hormonal stimulus. The lack of H3K4me3 at the TSS of these genes in aged UtSCs correlated with a striking lack of baseline expression and transcriptional activation. These effects were observed in the absence of dramatic changes in H3K4 methyltransferase or demethylase expression levels (Supplementary Figures 3 and 4A) (Hyun *et al.* 2017, Husmann & Gozani 2019). If anything, some of these H3K4 methyl writers were more highly expressed in aged UtSCs, in line with the net gain in H3K4me3-enriched sites (Fig. 4C) and the overall increase in mean expression levels of DE genes (Fig. 2D). Although not distinguished by specific GO terms, this set of decidualization-induced H3K4me3-high genes may hold the key to the deficits observed in aged UtSCs. Intriguingly, *Epha3* is a marker of endometrial mesenchymal stem or progenitor cells, a population that has been mapped to the perivascular niche (To *et al.* 2014, Cousins *et al.* 2021). Similarly, *Gata5* is known for its role in microvascular endothelial cells, and gene ablation leads to vascular endothelial dysfunction (Reiter *et al.* 1999, Messaoudi *et al.* 2015). These deficits point to a specific deficit in the endometrial perivascular niche, which harbors mesenchymal stem-like progenitor cells required for normal uterine function. This view is strongly corroborated by the lower expression levels of the best-known endometrial mesenchymal stem cell marker, sushi domain containing 2 (*Susd2*), in aged UtSCs before and after decidualization (Supplementary Figure 4B) (Gargett *et al.* 2016, Gorsek Sparovec *et al.* 2022).

## Discussion

In the current study, we expand on our previous reports of a blunted decidualization response in the uterine stroma of aging mouse females (Woods *et al.* 2017, 2020). While an appropriate decidualization response of the uterine stroma upon embryo implantation depends on a continuous crosstalk between the luminal epithelium and the underlying endometrial stroma (Hantak *et al.* 2014), our previous data had suggested a specific failure of the stroma to induce P4-responsive genes such as *Prl8a2*, bone morphogenetic protein 2 (*Bmp2*) and secreted frizzled-related sequence protein 5 (*Sfrp5*). These conclusions were based on a small number of candidate genes assessed in isolated UtSCs. In this study, we assessed the transcriptional failings that occur in UtSCs as a function of maternal age on the global level before and after induction of decidualization by hormone exposure *in vitro*. We combined these transcriptomic data with epigenetic profiling for two key histone modifications, H3K4me3 and H3K9me3, representing well-established active and repressive marks, respectively.

Our data demonstrate that age imposes by far the greatest transcriptomic differences on UtSCs. However, while the young cells are clearly transcriptionally distinct on the global scale before and after hormone treatment, the aged samples remain overall very similar to each other regardless of hormone exposure. These data demonstrate a fundamental deficiency of aged UtSCs to respond to the pregnancy hormone stimulus. This deficit will greatly enhance the risk of pregnancy complications, as corroborated by the observed increase in the number of fetuses displaying developmental defects in litters to older mouse females (Lopes *et al.* 2009, Woods *et al.* 2017).

Gene categories that change the most with age broadly relate to cell adhesion, extracellular matrix components, vascular as well as muscle biology. These processes are all consistent with expectations for endometrial stromal cell function. Indeed, an increase in collagen fibers, a hallmark of aging, has been reported over 60 years ago (Biggers et al. 1962*a*, Kong *et al.* 2012). Similarly, vessel elasticity is known to decline with age (Xu *et al.* 2017, Gonzalez *et al.* 2023). What was surprising in our data, however, was a particular enrichment of certain endothelial cell marker genes in aged UtSCs, such as platelet/endothelial cell adhesion molecule 1 (*Pecam1* a.k.a* Cd31*), multiple arterial and venous markers, as well as the majority of lymphatic markers. Yet, these genes were not equally dysregulated in bulk RNA-seq data of aged uteri *in vivo*, and neither did we find evidence for a denser vascular bed in aged uteri as measured by immunostaining (data not shown). Thus, we are left with multiple possible scenarios to explain these findings: Perhaps these gene expression changes are masked in bulk tissue RNA-seq due to a small number of cells being affected, suggesting that single-cell RNA sequencing approaches might help to resolve this possibility. Alternatively, it is conceivable that the changes in extracellular matrix composition with age favor the isolation of particular vascular cell types with the enzymatic digestion cocktail used for the isolation of UtSCs, such that the apparent enrichment of certain vascular marker genes may be the result of the technical procedures applied to an altered tissue context. Finally, and perhaps more intriguingly, it is possible that the tight control of gene regulation decreases with age, allowing for the mis-expression of numerous genes when cells are taken out of their tissue context. Such an increase in transcriptional variation and associated loss of transcriptional fidelity is indeed a well-known effect of organismal aging (Bahar *et al.* 2006, Southworth *et al.* 2009, Martinez-Jimenez *et al.* 2017, Nikopoulou *et al.* 2019). This possibility would also explain why some vascular genes are up-regulated while others are down-regulated in aged UtSCs. In any case, these data suggest that aged uteri are more prone to gene mis-regulation and that this enhanced susceptibility preferentially affects vascular and muscle-related genes, both of which fundamentally govern uterine function during pregnancy.

A particular novelty of our study is the epigenomic profiling approach of UtSCs in ‘ground state’ conditions, i.e. in the absence of a decidualization stimulus. This approach reveals whether UtSCs of aged females are already epigenetically distinct., which in turn may predispose them to fail in their differentiation response. This is indeed what we observed. Thus, UtSCs of aged females were identifiable based on their histone modification profiles alone, exhibiting stark differences compared to the UtSCs from young females. These epigenetic changes were particularly pronounced for H3K4me3, with around 15% of H3K4me3 peaks significantly altered in enrichment upon age, which correlates with the widespread expression differences observed. Although the changes in H3K9me3 distribution were less pronounced, as perhaps expected, they still resulted in a clear epigenetic distinction between young and aged UtSCs. We identified numerous genes in which the gain in H3K4me3 correlated with elevated expression levels as well as with a concomitant loss of H3K9me3 at the same gene loci; conversely, other loci displayed a loss of H3K4me3 and concomitant gain in H3K9me3 and lowered transcriptional output. While these correlations hold up, it is noteworthy that the H3K9me3 peaks most commonly do not overlap with the H3K4me3 peaks at gene promoters. Thus, whether or not the altered H3K9me3 abundance is causatively related to transcriptional changes is arguable and would only hold up if they affected an enhancer element or transcription factor binding site. Future experiments may therefore call for the profiling of the polycomb mark H3K27me3 that, together with H3K4me3, forms a bivalent epigenetic signature at genes poised for activation in embryonic stem cells (Azuara *et al.* 2006, Bernstein *et al.* 2006). The limited amount of material that can be obtained from one uterus, combined with the significantly reduced proliferative capacity of UtSCs from aged females (Woods *et al.* 2017), prevented us from studying this third mark from the same set of mice. Instead, our preference in this study was for H3K9me3 as a mark of constitutive heterochromatin in our efforts to identify gross epigenomic changes with age in UtSCs.

The combination of epigenetic and transcriptional profiling approaches revealed a dual set of shortcomings in aged UtSCs that will chiefly contribute to the often catastrophic deficits in the progression of decidualization exhibited by these cells. First, we identify a distinct failure in the up-regulation of genes that normally strongly respond to the steroid hormones E2 and P4 (Fig. 3E). Second it would appear that these genes need to be already heavily marked by H3K4me3 in undifferentiated conditions in order to be further up-regulated, and – perhaps – to prevent the stochastic accumulation of DNA methylation (Okitsu and Hsieh 2007, Bogdanovic *et al.* 2011, Balasubramanian *et al.* 2012, Rose and Klose 2014). Many of the genes that fail to become transcriptionally up-regulated distinguish themselves by already lacking distinct H3K4me3 peaks in undifferentiated conditions (Fig. 5E). This combination of failings identifies key regulators of endometrial biology, including important ion channels (De Clercq *et al.* 2015). Perhaps most intriguingly, however, it highlights a deficit in the perivascular niche compartment that harbors endometrial mesenchymal stem cells that are important for the regeneration of the endometrium but that are also capable of undergoing decidualization themselves (Gorsek Sparovec *et al.* 2022). This finding is further underpinned by the widespread dysregulation of genes broadly categorized as vascular-related that have been discussed above.

## Conclusion

Taken together, in this study, we provide a detailed characterization of the decidualization deficits displayed by uterine endometrial stromal cells as a consequence of advanced maternal age. Our transcriptomics analysis highlights a broad impedance in decidualization, which we tie to the distinct mis-regulation of key histone marks at genes with critical functions in uterine biology. These data provide important insights into the gene regulatory changes that occur in the aging uterus and that underlie the significant increase in pregnancy complications observed in females of advanced maternal age.

## Supplementary Materials

Supplementary Material

## Declaration of interest

The authors declare that there is no conflict of interest that could be perceived as prejudicing the impartiality of the study reported.

## Funding

This work was supported by the Biotechnology and Biological Sciences Research Councilhttp://dx.doi.org/10.13039/501100000268 (BBSRC) Strategic Programme Grant BB/J004499/1, by the Canadian Institutes of Health Researchhttp://dx.doi.org/10.13039/501100000024 project grant RN435448 – 450828 to MH and WD, by a Tier I Canada Research Chair in Developmental Genetics and Epigenetics (CRC-2018-00240) to MH, by NSERC Discovery Grant RGPIN-2021-02417 to MH, and by the Alberta Children’s Hospital Research Institute.

## Author contribution statement

LW performed the primary experiments, LW and MH performed data analyses, MH generated figures, and WD and MH conceived the project, obtained funding, and interpreted the data.
