# Supplementary material for: REPRODUCTIVE AGEING: Altered histone modification landscapes underpin defects in uterine stromal cell decidualization in aging females
Source: Reproduction. 2024 Aug 2;168(3):e240171. doi: 10.1530/REP-24-0171 (PMC11378140; doi:10.1530/REP-24-0171)
Supplement: Supplementary Material [file supplementary_material.pdf]

Suppl. Figure 1

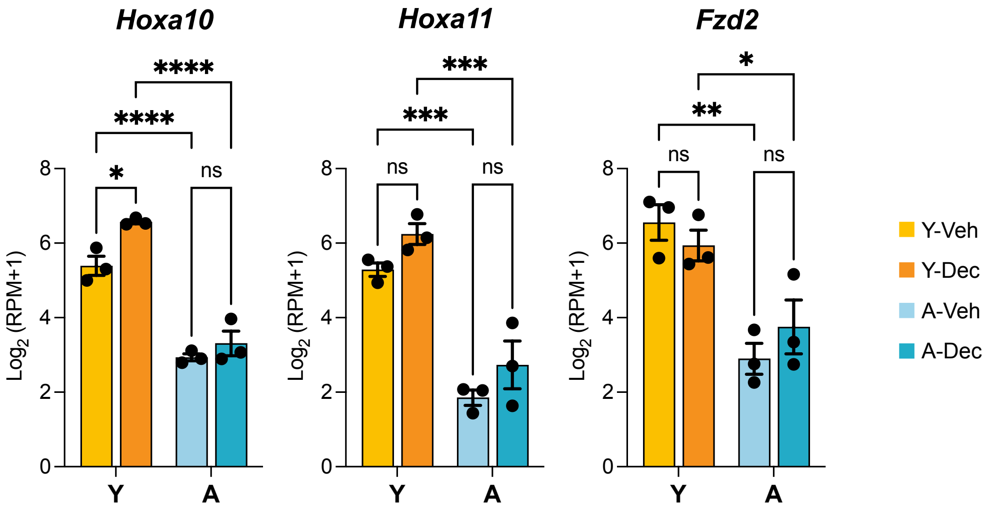

Supplementary Figure 1. Expression of DNA methylated genes is lower in aged UtSCs. Expression levels as determined by RNA-seq (Log_2 (RPM+1)) for genes that had been previously identified as being more highly DNA methylated in mouse uterine tissue of aged females (Woods, et al. 2020). Higher DNA methylation levels at specific genes in uterine tissue of aged females correlates with lower expression levels of these genes in the stromal cell compartment. Statistical significance analysis was performed by 2-way ANOVA.

Suppl. Figure 2

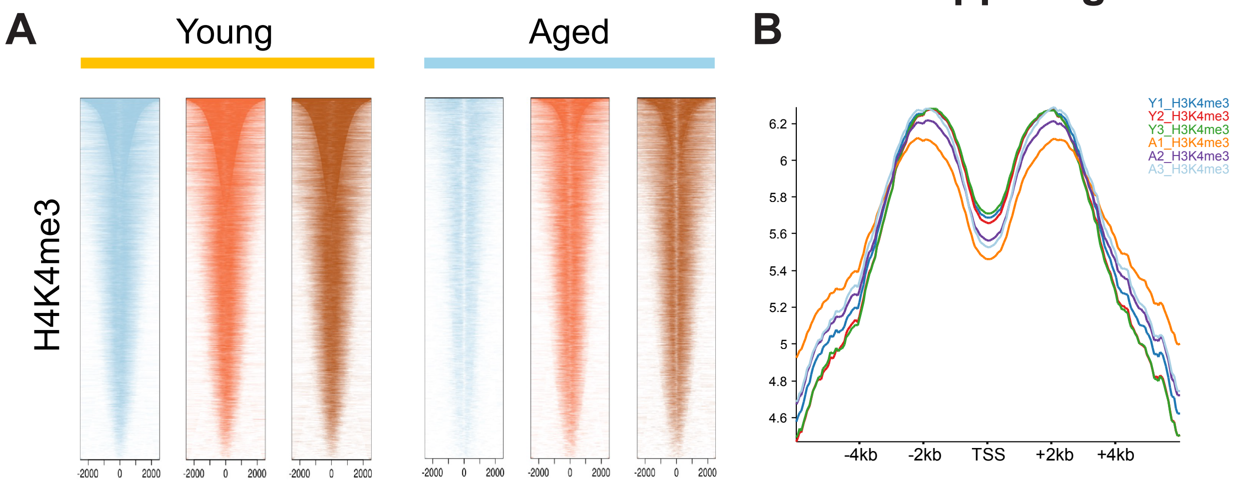

Supplementary Figure 2. H3K4me3 peak distribution.
(**A**) H3K4me3 ChIP-seq enrichment centered -2.5kb to +2.5kb around transcriptional start sites (TSSs) of genes. (**B**) Line graph of the characteristic bimodal distribution of H3K4me3 around the TSS. Since the TSS is nucleosome-depleted, promoter-associated H3K4me3 usually displays a “dip” in enrichment over the immediate TSS.

Suppl. Figure 3

H3K4 methyltransferase expression

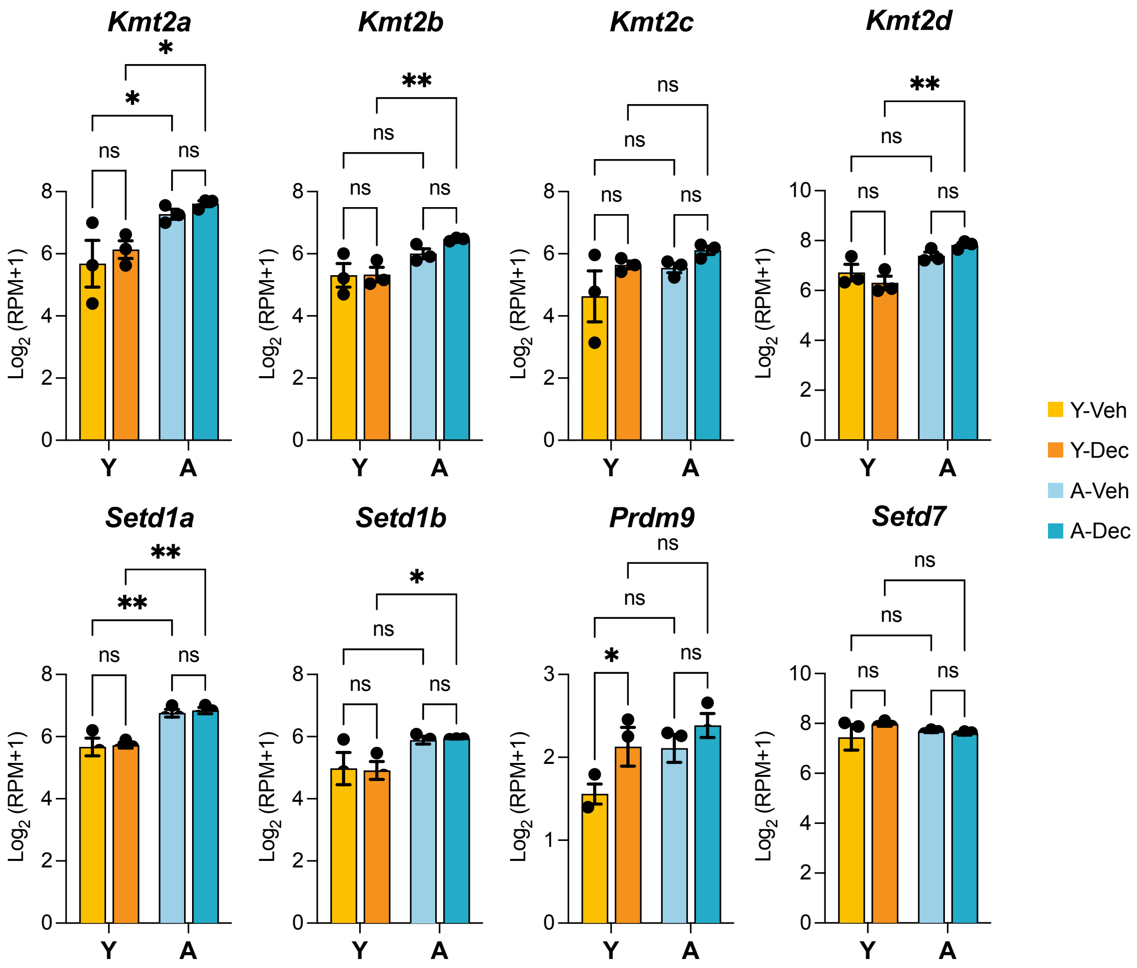

Supplementary Figure 3. Gene expression dynamics of histone H3 lysine 4 methyltransferases. Expression levels as determined by RNA-seq of the various histone H3K4 methyltransferases in young and aged UtSCs with or without hormone treatment. Statistical significance analysis was performed by 2-way ANOVA.

Suppl. Figure 4

A

H3K4 demethylase expression

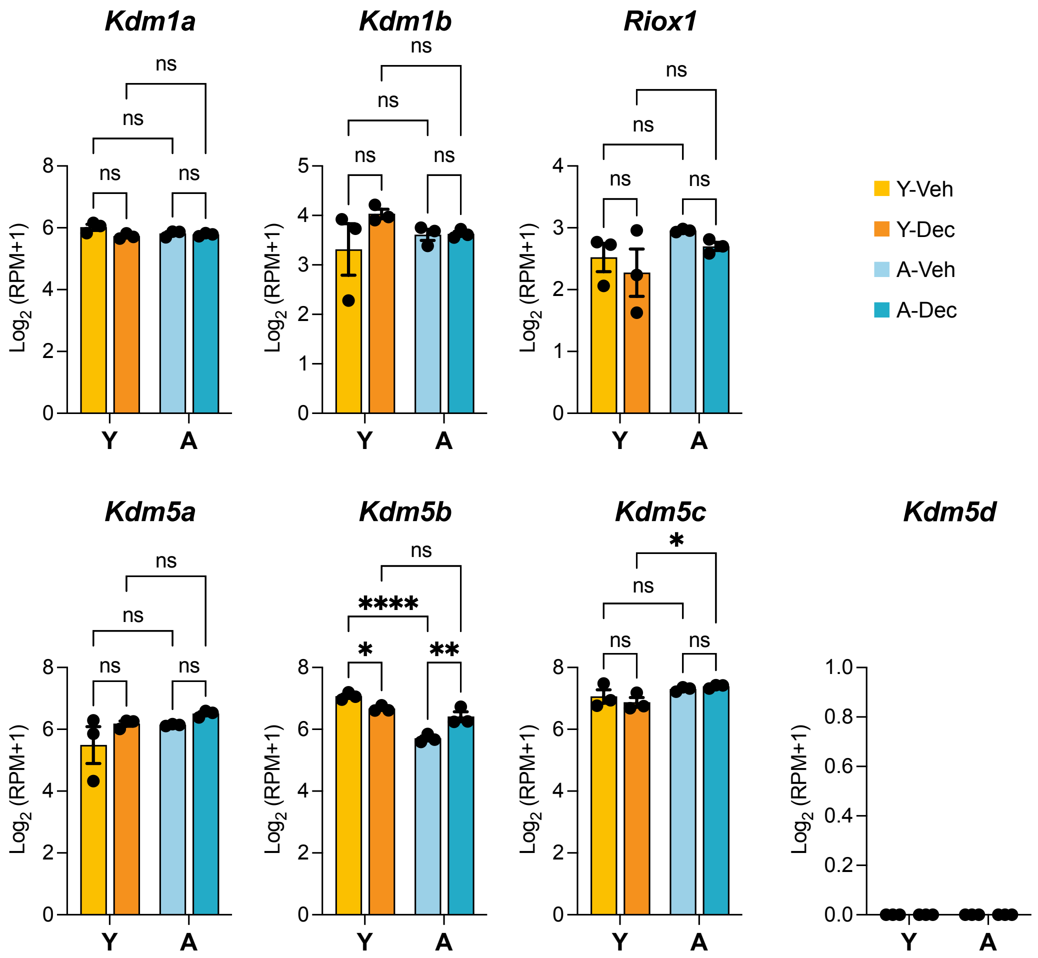

B

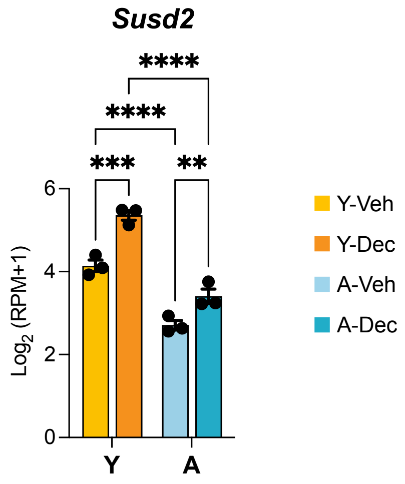

Supplementary Figure 4. Gene expression dynamics of histone H3 lysine 4 demethylases and of mesenchymal stem cell marker *Susd2*.

(**A**) Expression levels as determined by RNA-seq of the various histone H3K4 demethylases in young and aged UtSCs with or without hormone treatment. Statistical significance analysis was performed by 2-way ANOVA. (**B**) Expression dynamics of the mesenchymal stem cell marker *Susd2* in the same set of samples. Statistical significance analysis was performed by 2-way ANOVA.

Supplementary Table 1

Young-enriched H3K4me3-marked decidualization genes

Gene	Chr	Start	End	ID	Y1_V	Y2_V	Y3_V	Y1_D	Y2_D	Y3_D	A1_V	A2_V	A3_V	A1_D	A2_D	A3_D
Sntg1	1	8361475	9299878	ENSMUSG00000025909	1.71	1.06	1.96	3.44	4.04	3.63	0.10	0.00	0.00	0.77	0.00	0.28
Adhfe1	1	9547948	9580673	ENSMUSG00000025911	1.35	1.52	2.56	3.54	3.49	3.24	0.74	1.07	0.19	1.00	0.20	0.83
Pi15	1	17601901	17630939	ENSMUSG00000067780	6.75	5.21	6.70	8.18	8.43	7.95	1.48	2.14	2.09	3.58	4.69	4.22
Col3a1	1	45311538	45349706	ENSMUSG00000026043	10.86	11.03	11.05	11.94	11.75	12.07	5.18	6.94	5.91	6.49	10.05	7.60
Tmeff2	1	50900647	51187270	ENSMUSG00000026109	3.96	3.82	4.31	5.74	5.21	5.53	1.13	0.77	0.77	1.54	1.53	1.47
Glb1l	1	75198236	75210813	ENSMUSG00000026200	4.03	3.11	3.68	4.83	4.44	4.89	2.65	2.71	2.54	3.53	3.53	3.28
Col4a3	1	82586921	82722059	ENSMUSG00000079465	2.84	1.19	2.66	3.76	4.65	4.22	0.46	0.68	0.28	0.85	0.46	0.10
Ugt1a10	1	88055388	88219004	ENSMUSG00000090165	5.75	6.09	6.36	7.35	7.00	7.13	4.18	4.88	3.90	4.24	5.79	4.31
Ugt1a9	1	88070800	88218997	ENSMUSG00000090175	5.75	6.09	6.36	7.35	7.00	7.13	4.18	4.88	3.90	4.24	5.79	4.31
Ugt1a8	1	88087813	88219004	ENSMUSG00000089675	5.75	6.09	6.36	7.35	7.00	7.13	4.18	4.88	3.90	4.24	5.79	4.31
Ugt1a7c	1	88095062	88220002	ENSMUSG00000090124	6.88	6.50	7.54	8.65	8.61	8.09	5.15	5.79	4.88	5.18	6.70	5.22
Ackr3	1	90203980	90216751	ENSMUSG00000044337	3.44	2.77	3.12	4.89	5.30	4.93	2.30	2.68	1.77	2.30	2.45	2.97
Col6a3	1	90765923	90843971	ENSMUSG00000048126	8.61	7.58	8.40	9.18	9.98	9.67	4.88	6.17	6.02	6.44	9.15	7.71
Ramp1	1	91179822	91225196	ENSMUSG00000034353	3.21	4.14	2.72	5.31	4.43	5.25	0.60	0.14	0.77	1.20	1.41	1.09
Ildr2	1	166254139	166316823	ENSMUSG00000040612	5.90	4.98	5.95	7.51	7.36	7.49	2.70	2.46	2.30	2.79	3.44	2.68
Itga8	2	12106632	12301922	ENSMUSG00000026768	6.27	4.40	5.60	7.71	7.81	7.46	2.63	2.11	2.07	4.26	5.42	3.91
Cercam	2	29869164	29882840	ENSMUSG00000039787	6.17	6.33	5.61	7.19	6.84	7.45	2.75	2.95	2.61	3.61	4.28	3.38
Ptgs1	2	36230426	36252272	ENSMUSG00000047250	6.54	6.07	6.32	7.19	7.02	7.47	3.65	4.27	3.87	3.66	4.50	4.15
Fmnl2	2	52857860	53133804	ENSMUSG00000036053	3.98	2.28	4.04	5.21	5.52	4.98	6.19	5.89	6.42	7.21	6.53	7.35
Dpp4	2	62330073	62412231	ENSMUSG00000035000	4.56	4.48	5.48	6.49	7.30	6.67	0.92	0.26	0.36	0.00	0.11	0.10
Cobll1	2	65088339	65239403	ENSMUSG00000034903	5.72	3.86	5.89	6.99	7.25	7.19	6.03	5.94	5.74	5.82	6.12	5.94
Olfr1033	2	86020633	86045171	ENSMUSG00000045392	2.58	1.42	1.87	4.03	4.08	4.58	0.00	0.00	0.00	0.38	0.30	0.36
Ptprj	2	90429754	90580647	ENSMUSG00000025314	4.94	3.52	4.01	5.35	5.66	5.59	3.93	4.27	2.97	3.87	5.03	3.57
Fibin	2	110360917	110363183	ENSMUSG00000074971	4.82	4.75	5.18	7.78	7.85	7.64	2.63	2.93	2.65	4.00	5.12	4.14
Fgf7	2	126034658	126091185	ENSMUSG00000027208	2.73	1.79	3.02	4.77	5.53	4.60	2.26	2.17	2.42	3.60	4.63	4.44
Gata5	2	180325133	180334699	ENSMUSG00000015627	3.56	3.73	3.73	5.24	4.45	5.69	0.38	0.26	0.58	1.20	0.61	0.71
Pcdh10	3	45378398	45435623	ENSMUSG00000049100	0.24	0.00	0.55	2.06	1.47	2.17	0.00	0.00	0.00	0.77	0.61	0.51
Trpc4	3	54156035	54318471	ENSMUSG00000027748	3.25	3.20	2.81	5.51	5.28	5.21	0.92	0.68	1.05	1.59	2.30	1.70
Smad9	3	54755582	54801257	ENSMUSG00000027796	4.49	3.11	4.31	5.25	5.28	5.28	3.14	3.60	2.32	3.59	4.24	3.33
Mme	3	63241537	63386030	ENSMUSG00000027820	1.00	0.37	1.11	5.65	6.35	5.26	0.60	0.00	0.00	1.07	0.30	0.44
Kcnab1	3	64949196	65378223	ENSMUSG00000027827	4.78	3.33	4.78	5.87	5.91	6.45	3.57	3.09	2.63	3.75	2.91	2.06
Ctsk	3	95499256	95509362	ENSMUSG00000028111	3.92	4.31	4.56	5.73	5.48	5.54	2.07	2.81	2.66	3.40	5.04	3.59
Pde4dip	3	97689824	97888707	ENSMUSG00000038170	8.54	7.04	7.86	9.10	8.81	9.38	5.90	5.95	6.07	6.34	7.18	6.54
Tent5c	3	100451628	100489324	ENSMUSG00000044468	3.02	2.85	2.75	8.33	8.31	8.22	1.40	1.38	0.71	1.93	3.53	2.55
Gm12474	3	100489664	100523920	ENSMUSG00000053957	0.00	0.00	0.00	1.77	1.42	1.72	0.00	0.00	0.00	0.00	0.00	0.00
Olfml3	3	103722222	103738001	ENSMUSG00000027848	6.19	6.59	6.50	9.46	9.17	9.16	1.93	2.41	2.34	2.59	4.09	3.06
Hipk1	3	103739815	103791563	ENSMUSG00000008730	7.08	5.24	6.25	7.36	7.88	7.71	8.07	7.90	8.32	8.75	8.32	8.79
Col11a1	3	114030540	114220718	ENSMUSG00000027966	8.23	7.35	8.14	10.83	11.19	11.11	5.86	5.98	5.31	7.38	9.24	7.40
Usp53	3	122931493	122984510	ENSMUSG00000039701	5.19	3.28	5.13	6.57	7.27	6.85	3.78	3.54	3.64	4.69	4.65	4.80
Asph	4	9448069	9669344	ENSMUSG00000028207	7.22	5.86	6.85	8.26	8.28	8.23	5.89	5.92	6.07	5.99	6.32	6.11
Frem1	4	82897920	83052339	ENSMUSG00000059049	4.97	2.31	3.62	6.27	6.28	6.59	0.53	0.93	0.51	1.20	3.68	1.54
Hacd4	4	88396144	88438928	ENSMUSG00000028497	7.26	6.65	7.34	8.13	8.16	7.92	6.07	5.58	5.57	5.57	5.88	5.17
Acot11	4	106744555	106804998	ENSMUSG00000034853	5.84	5.11	5.54	6.76	6.25	6.96	3.15	2.49	2.38	3.58	3.66	3.59
Smim1	4	154020470	154026230	ENSMUSG00000078350	6.17	6.26	6.38	7.73	7.33	7.87	2.17	2.17	1.86	2.93	4.37	3.35
Steap4	5	7960457	7982213	ENSMUSG00000012428	1.45	0.48	1.78	2.67	2.82	2.61	0.29	0.59	0.58	0.26	0.61	0.44
Gnai1	5	18265135	18360355	ENSMUSG00000057614	3.46	3.02	3.93	4.79	4.51	4.83	1.18	0.85	0.77	1.27	0.81	1.50
Magi2	5	19227036	20704792	ENSMUSG00000040003	3.41	1.95	2.78	4.07	4.00	4.15	0.97	1.00	0.65	1.77	1.78	1.47
Fras1	5	96373955	96784728	ENSMUSG00000034687	3.85	3.00	3.42	5.41	5.71	6.29	0.97	0.77	0.77	1.44	2.06	0.77
Plac8	5	100553725	100572245	ENSMUSG00000029322	3.44	4.04	3.47	4.47	5.58	4.59	2.80	3.76	2.84	3.62	5.29	3.95
Tgfbr3	5	107106570	107289629	ENSMUSG00000029287	6.66	5.43	6.33	7.49	7.75	7.71	6.04	6.17	6.57	6.97	6.51	7.35
Tmem119	5	113793729	113800516	ENSMUSG00000054675	6.25	6.00	4.55	7.05	6.33	8.22	0.97	2.38	1.32	1.93	4.23	3.14
Upk3b	5	136038496	136046487	ENSMUSG00000042985	7.52	7.68	7.68	9.83	9.60	10.40	1.59	1.33	0.71	2.56	2.41	1.73
Gm20488	5	149368476	149430682	ENSMUSG00000107314	4.55	3.22	4.16	6.52	6.88	6.81	1.72	2.24	2.53	2.27	3.99	2.67
Medag	5	149411749	149431723	ENSMUSG00000029659	5.38	5.59	5.78	7.97	8.27	7.76	2.75	3.25	3.45	3.04	4.99	3.86
Col1a2	6	4504814	4541544	ENSMUSG00000029661	11.64	11.80	11.57	12.90	12.59	13.01	6.49	6.46	6.57	8.44	9.86	8.43
Pon3	6	5220852	5256286	ENSMUSG00000029759	5.12	4.15	4.73	6.04	5.86	6.23	4.39	4.59	4.41	4.17	4.62	3.96
Cntnap2	6	45059357	47304213	ENSMUSG00000039419	0.45	0.26	1.62	2.43	2.88	2.50	0.00	0.00	0.00	0.00	0.20	0.00
Mpp6	6	50110241	50198939	ENSMUSG00000038388	5.59	6.04	6.34	8.48	8.44	7.76	5.62	5.44	5.77	4.83	4.51	5.32
Hoxa10	6	52231197	52240854	ENSMUSG00000000938	5.87	5.00	5.30	6.50	6.68	6.53	3.11	2.79	2.90	2.89	3.97	3.07
Klf15	6	90462576	90475238	ENSMUSG00000030087	1.40	1.42	0.49	3.74	3.23	3.89	0.46	0.00	0.10	2.35	1.64	1.88
A730049H05Rik	6	92816478	92847174	ENSMUSG00000048636	2.95	2.45	3.01	5.61	5.71	5.61	0.10	0.26	0.00	0.00	0.54	0.10
Frmd4b	6	97286867	97617541	ENSMUSG00000030064	5.68	4.60	5.76	7.13	6.95	7.00	5.71	5.48	5.48	5.96	5.64	5.63
Cntn6	6	104492790	104863406	ENSMUSG00000030092	0.00	0.00	0.00	0.91	0.79	0.43	0.00	0.00	0.00	0.00	0.00	0.00
Il17re	6	113458484	113470758	ENSMUSG00000043088	3.93	4.01	3.42	5.03	4.47	5.33	0.00	0.00	0.00	0.26	0.00	0.28
Slc2a3	6	122727809	122801640	ENSMUSG00000003153	3.89	4.03	3.55	4.57	4.71	5.20	0.29	0.85	0.28	1.07	1.10	0.99
C1s1	6	124530345	124542359	ENSMUSG00000038521	8.66	7.84	8.40	9.41	9.35	9.48	3.56	4.39	3.28	3.79	4.60	3.90

Ldhb	6	142490249	142507957	ENSMUSG00000030246	6.05	7.10	6.66	8.34	7.88	7.85	4.97	5.35	4.55	4.69	5.21	4.58
Sox5	6	143828425	144781977	ENSMUSG00000041540	4.05	2.75	3.70	4.94	5.13	5.08	1.96	2.14	1.58	3.09	3.73	2.55
Bicd1	6	149408886	149563329	ENSMUSG00000003452	4.81	3.96	4.97	6.77	7.01	7.03	2.96	2.04	1.86	3.21	2.24	2.11
Saa3	7	46711998	46715700	ENSMUSG00000040026	5.33	6.67	5.03	7.49	7.39	7.64	0.92	1.21	0.71	0.00	0.61	0.44
Atp10a	7	58656166	58829420	ENSMUSG00000025324	6.97	5.76	6.19	7.81	7.53	8.03	5.40	5.16	5.44	4.73	4.33	4.75
Ctsc	7	88278085	88310888	ENSMUSG00000030560	6.31	6.70	6.64	7.93	8.00	7.42	4.66	5.83	4.38	4.42	5.93	4.44
Dlg2	7	90476672	92449247	ENSMUSG00000052572	4.13	3.15	3.92	6.81	6.70	6.65	0.74	0.49	0.44	1.81	2.16	2.09
Rab30	7	92741603	92844535	ENSMUSG00000030643	3.47	3.25	3.73	4.47	4.58	4.46	3.80	3.45	3.04	3.84	4.04	3.12
Ppfibp2	7	107595207	107748583	ENSMUSG00000036528	2.37	2.71	2.78	3.76	4.02	3.89	1.51	2.27	0.89	1.14	1.64	0.83
Plekha7	7	116123485	116308376	ENSMUSG00000045659	4.35	3.06	3.52	5.68	5.23	6.09	1.62	1.21	1.40	2.11	1.74	1.39
Mosmo	7	120677618	120734854	ENSMUSG00000046096	5.17	4.62	5.09	5.86	6.22	5.93	4.87	4.57	4.62	5.01	4.93	4.85
Prrt2	7	127017531	127021211	ENSMUSG00000045114	2.93	2.06	2.49	3.30	3.92	3.67	0.46	1.38	1.44	1.27	1.74	1.70
Cpxm2	7	132032687	132154739	ENSMUSG00000030862	3.91	4.92	3.41	5.74	5.69	5.75	0.00	0.00	0.00	0.49	0.81	0.58
Ano1	7	144588549	144751974	ENSMUSG00000031075	0.86	1.13	0.43	3.04	2.73	2.91	2.93	0.68	1.28	2.21	0.61	0.65
Pdgfrl	8	40926212	40990785	ENSMUSG00000031595	3.05	3.42	3.31	5.11	4.36	4.79	1.27	1.07	1.23	1.07	1.04	1.31
Pdlim3	8	45885461	45919548	ENSMUSG00000031636	5.82	5.47	5.79	7.51	6.70	7.32	0.00	0.77	0.19	1.38	0.68	1.04
Hpgd	8	56294585	56321043	ENSMUSG00000031613	4.50	3.95	4.70	6.52	6.87	6.36	3.20	1.44	1.32	3.21	2.49	1.96
Hand2os1	8	57281117	57324233	ENSMUSG00000100510	4.91	4.41	4.98	6.36	6.51	6.20	0.92	0.68	1.05	1.85	1.37	1.79
Tox3	8	90247040	90348343	ENSMUSG00000043668	0.00	0.00	0.00	1.21	1.68	1.21	0.00	0.00	0.00	0.00	0.00	0.00
Ldhd	8	111623785	111630374	ENSMUSG00000031958	1.97	2.22	1.85	5.24	4.82	5.31	0.10	0.00	0.00	0.68	0.93	0.77
Crispld2	8	119992438	120052793	ENSMUSG00000031825	8.21	8.39	8.26	10.42	10.09	10.28	1.18	1.68	1.94	2.75	5.26	3.42
Kcnk1	8	125995170	126030685	ENSMUSG00000033998	1.97	1.52	2.63	3.92	3.45	4.03	0.00	0.00	0.00	0.00	0.54	0.19
Naalad2	9	18321951	18402995	ENSMUSG00000043943	4.58	4.20	5.04	5.97	5.96	5.59	0.74	0.26	0.89	1.64	1.98	1.93
Gramd1b	9	40293233	40531383	ENSMUSG00000040111	5.58	5.01	5.20	6.66	7.25	6.91	5.17	5.36	5.75	5.58	6.12	5.98
Usp2	9	44067021	44095627	ENSMUSG00000032010	3.67	3.40	3.02	4.83	4.52	5.09	3.25	2.83	3.09	2.85	2.85	2.75
Zbtb16	9	48654297	48836222	ENSMUSG00000066687	0.00	0.00	0.00	6.60	6.73	6.88	0.00	0.00	0.00	4.44	3.97	4.05
Trdn	10	33080554	33476709	ENSMUSG00000019787	4.70	5.76	5.67	6.84	7.19	6.32	0.67	0.00	0.28	0.77	0.20	0.28
Ank3	10	69398773	70027438	ENSMUSG00000069601	1.45	0.67	1.68	3.17	3.50	2.67	1.08	1.77	0.65	1.32	2.80	1.27
Fam13c	10	70440481	70558736	ENSMUSG00000043259	5.61	4.35	5.51	6.64	6.47	6.66	3.30	3.06	3.33	3.54	3.76	3.22
Suox	10	128669894	128674073	ENSMUSG00000049858	4.13	3.95	4.46	6.05	6.36	5.72	3.70	3.67	3.39	4.18	4.85	4.01
Adamts2	11	50602084	50807573	ENSMUSG00000036545	7.14	7.01	6.28	7.77	7.61	8.02	3.22	4.49	4.01	4.80	7.54	5.73
Gm2a	11	55098115	55113029	ENSMUSG00000000594	6.78	6.99	6.51	8.27	8.20	8.55	5.94	6.23	5.75	6.14	6.26	5.85
Arhgap44	11	65002039	65162961	ENSMUSG00000033389	4.85	3.72	4.22	5.70	5.33	6.21	3.88	4.07	3.93	4.33	4.13	4.26
Arl4d	11	101665541	101667832	ENSMUSG00000034936	3.72	3.47	2.82	4.61	4.39	5.57	1.03	1.27	0.71	1.27	1.74	0.99
Axin2	11	108920349	108950783	ENSMUSG00000000142	1.49	1.67	1.15	3.45	3.52	3.99	0.20	0.38	0.71	1.44	1.19	1.14
Sphk1	11	116530925	116536675	ENSMUSG00000061878	1.90	2.75	0.81	3.64	3.27	4.66	0.67	1.00	1.32	3.34	4.33	3.39
C1qtnf1	11	118428203	118449963	ENSMUSG00000017446	3.28	2.95	3.11	5.26	5.23	5.53	2.67	3.14	2.90	3.14	3.22	3.02
Cys1	12	24665833	24681813	ENSMUSG00000062563	1.35	1.91	2.21	3.42	3.83	3.64	0.10	0.49	0.19	1.54	2.74	1.39
Dact1	12	71309884	71320107	ENSMUSG00000044548	1.90	1.13	0.90	3.52	3.26	4.08	0.38	0.00	0.83	1.54	2.24	0.94
Rapgef5	12	117516479	117759737	ENSMUSG00000041992	2.06	1.25	1.57	6.03	6.06	5.75	4.46	3.83	4.56	5.55	5.40	5.94
Itgb8	12	119158022	119238802	ENSMUSG00000025321	3.49	1.47	2.89	4.36	4.43	4.76	1.48	1.85	1.05	3.94	4.05	3.47
Net1	13	3882018	3918220	ENSMUSG00000021215	6.02	5.35	6.49	9.21	9.36	9.19	5.95	5.98	5.75	6.25	5.41	5.85
B3galnt2	13	13954469	13999103	ENSMUSG00000039242	5.16	4.56	5.06	5.91	5.82	6.02	4.29	4.56	4.38	4.49	4.54	4.49
Omd	13	49582462	49592822	ENSMUSG00000048368	0.63	0.37	1.36	2.77	3.24	2.09	0.00	0.00	0.00	0.00	0.20	0.00
Adamts6	13	104287835	104496695	ENSMUSG00000046169	3.84	2.93	3.25	4.58	5.05	4.50	2.73	2.52	2.63	3.13	4.03	3.40
Itga2	13	114833081	114932100	ENSMUSG00000015533	5.37	4.33	5.38	7.13	8.12	7.22	2.19	3.37	2.68	3.04	4.63	3.71
Usp54	14	20548912	20641063	ENSMUSG00000034235	5.46	4.13	5.21	6.25	6.32	6.52	5.54	5.22	5.40	6.14	5.36	5.87
Oxnad1	14	32085374	32103202	ENSMUSG00000021906	3.91	4.22	3.72	4.85	4.82	5.51	3.44	3.01	3.13	3.00	3.06	2.83
Armh4	14	49675952	49783383	ENSMUSG00000036242	2.37	2.42	2.49	4.81	4.77	4.51	0.00	0.14	0.00	0.00	0.46	0.83
Pnp	14	50931082	50965237	ENSMUSG00000115338	5.45	6.65	5.77	7.60	7.25	7.49	6.65	6.80	6.82	7.07	6.51	6.98
Gm49342	14	50944517	50963136	ENSMUSG00000021871	4.32	4.60	4.29	6.39	6.12	6.13	5.27	5.35	5.46	5.71	5.20	5.64
Prlr	15	10177238	10349180	ENSMUSG00000005268	1.49	0.99	1.48	3.62	3.99	3.40	0.00	0.00	0.00	0.00	0.20	0.10
Adcy8	15	64697084	64922296	ENSMUSG00000022376	1.18	0.00	0.08	2.46	2.40	2.45	0.29	0.38	0.19	1.49	1.04	1.09
Cyp2d22	15	82370527	82380260	ENSMUSG00000061740	3.89	4.48	4.01	5.47	5.53	5.64	1.31	2.04	2.32	1.97	3.26	3.19
Cpne8	15	90487482	90679432	ENSMUSG00000052560	4.89	5.31	5.85	7.21	7.12	6.30	5.01	4.88	5.23	5.15	4.72	5.35
BC106179	16	23220656	23225484	ENSMUSG00000045231	2.16	0.76	1.51	2.94	2.61	3.20	0.29	0.68	0.44	1.00	0.87	0.28
St6gal1	16	23224740	23360350	ENSMUSG00000022885	6.04	5.41	5.84	7.09	7.01	7.27	4.07	4.49	3.94	5.09	5.14	4.84
Itgb5	16	33829665	33949338	ENSMUSG00000022817	7.92	8.20	7.32	9.23	8.42	9.26	4.86	5.84	4.96	6.21	7.63	6.06
Upk1b	16	38773184	38800328	ENSMUSG00000049436	5.78	5.86	6.80	8.87	8.50	8.73	1.91	1.89	1.28	2.91	2.88	2.09
Boc	16	44485049	44558897	ENSMUSG00000022687	5.77	5.11	5.17	6.54	6.38	6.88	3.10	3.61	3.23	3.52	4.94	3.49
Epha3	16	63543534	63864175	ENSMUSG00000052504	3.92	2.93	3.69	4.80	4.65	4.53	0.00	0.00	0.00	0.85	1.10	0.19
Smoc2	17	14279506	14404790	ENSMUSG00000023886	7.46	7.18	7.04	8.86	8.67	8.96	3.21	5.11	4.39	3.65	6.15	5.06
Sox8	17	25565892	25570686	ENSMUSG00000024176	1.18	0.76	0.49	1.90	1.81	2.43	0.29	0.14	0.00	0.26	0.38	0.00
Msln	17	25748613	25754378	ENSMUSG00000063011	7.86	8.17	7.49	10.22	9.74	10.62	0.53	1.33	0.71	2.77	2.89	2.37
Daam2	17	49456022	49564343	ENSMUSG00000040260	6.67	6.31	6.48	7.80	7.46	7.93	1.08	1.21	0.71	2.17	3.56	1.91
Gata6os	18	10987944	11052567	ENSMUSG00000097222	3.00	2.85	2.83	4.14	3.73	4.16	1.48	1.59	1.23	1.81	2.19	2.27
Celf4	18	25477632	25754157	ENSMUSG00000024268	3.98	3.97	3.93	4.99	4.87	4.77	2.63	2.27	2.32	1.68	1.87	1.76
Lvrn	18	46850038	46907242	ENSMUSG00000024481	5.20	5.12	5.52	6.46	6.40	6.31	2.90	3.64	3.66	4.41	3.94	4.89
Lox	18	52516067	52529867	ENSMUSG00000024529	10.65	9.45	10.59	11.86	12.32	12.01	6.47	7.06	6.19	8.40	9.82	8.28
Isoc1	18	58659466	58680368	ENSMUSG00000024601	6.40	6.13	6.36	7.89	8.06	7.68	4.16	3.86	4.44	3.46	4.01	3.72
Rab27b	18	69979131	70141605	ENSMUSG00000024511	2.58	1.99	3.29	5.10	4.94	4.53	3.40	3.30	2.23	3.56	3.45	2.20
Myo5b	18	74440936	74771493	ENSMUSG00000025885	2.45	2.79	1.87	4.09	4.12	4.35	0.20	0.26	0.36	1.00	0.54	1.50
Acaa2	18	74779197	74806208	ENSMUSG00000036880	5.02	6.24	5.44	7.42	7.00	7.34	4.07	4.21	4.00	4.00	4.31	4.04

Neto1	18	86394952	86506718	ENSMUSG00000050321	1.35	0.84	1.45	2.86	2.92	2.67	0.00	0.00	0.10	0.00	0.00	0.19
Prune2	19	16956118	17223932	ENSMUSG00000039126	5.94	4.28	4.87	6.43	6.65	6.71	2.72	2.14	2.63	3.17	3.95	3.52
Aldh1a1	19	20492715	20643465	ENSMUSG00000053279	4.41	4.62	5.33	9.37	9.22	9.02	1.76	1.27	1.32	5.46	5.15	4.19
Klf9	19	23141226	23168134	ENSMUSG00000033863	6.35	4.35	5.93	7.16	7.53	7.29	3.95	3.92	4.31	5.96	5.83	5.90
Plce1	19	38481109	38785030	ENSMUSG00000024998	2.79	2.12	2.78	3.62	4.01	4.00	1.08	0.38	1.83	1.32	1.15	2.50
Ablim1	19	57032733	57314919	ENSMUSG00000025085	4.95	4.24	4.65	6.37	6.44	6.79	8.17	7.65	8.18	8.50	7.35	8.31
Srpx	X	10037977	10117709	ENSMUSG00000090084	6.06	5.81	5.91	6.85	6.76	6.82	0.92	1.00	0.71	0.77	3.03	1.61
Gria3	X	41400854	41678601	ENSMUSG00000001986	6.68	5.74	7.15	8.12	8.14	7.78	5.57	5.25	5.07	5.68	5.80	5.24
Prrg3	X	71962624	71972722	ENSMUSG00000033361	4.03	3.44	3.91	6.46	6.15	6.01	0.92	1.49	1.19	0.77	1.04	1.47
Gabra3	X	72432681	72656848	ENSMUSG00000031343	3.87	3.59	4.24	7.04	6.99	6.66	0.10	0.00	0.83	0.38	0.68	0.77
